# Mechanical thrombectomy combined with intravenous thrombolysis for acute ischemic stroke: a systematic review and meta-analyses

**DOI:** 10.1038/s41598-023-35532-7

**Published:** 2023-05-26

**Authors:** Meiling Zheng, Li Li, Lizhou Chen, Bin Li, Cuiling Feng

**Affiliations:** 1 Dongzhimen Hospital, Beijing University of Chinese Medicine, Beijing, 100010 People’s Republic of China; 2 Department of Radiology, Hospital of Chengdu University of Traditional Chinese Medicine, Chengdu, 610072 Sichuan Province People’s Republic of China; 3 Department of Radiology, West China Hospital of Sichuan University, Chengdu, 610041 Sichuan Province People’s Republic of China; 4 Department of Geriatrics, Hospital of Chengdu University of Traditional Chinese Medicine, Chengdu, 610072 People’s Republic of China; 5 Present Address: Peking University People’s Hospital, Beijing, 100000 People’s Republic of China

**Keywords:** Diseases of the nervous system, Neuro-vascular interactions

## Abstract

To assess the clinical value of mechanical thrombectomy (MT) combined with intravenous thrombolysis (IVT) in acute ischemic stroke (AIS) by comparing it with the MT alone. In this study, we conducted a comprehensive meta-analysis of both observational and randomized controlled studies (RCTs) to investigate various outcomes. Our search for relevant studies was conducted between January 2011 and June 2022 in four major databases: PubMed, Embase, WOS, and Cochrane Library. We collected data on several outcomes, including functional independence (FI; defined as modified Rankin Scale score of 0 to 2), excellent outcomes (mRS 0–1), successful recanalization (SR), symptomatic intracerebral hemorrhage (sICH), any intracerebral hemorrhage (aICH), and mortality at three months or discharge. The primary efficacy outcome and safety outcome were FI and sICH, respectively, whereas excellent outcomes and SR were considered secondary efficacy outcomes. Additionally, mortality and aICH were analyzed as secondary safety outcomes. We employed the Mantel–Haenszel fixed-effects model for RCTs when *I*^2^ < 50%, otherwise the random-effects model was utilized. For observational studies and subgroup analyses, we used the random-effects model to minimize potential bias. A total of 55 eligible studies (nine RCTs and 46 observational studies) were included. For RCTs, the MT + IVT group was superior in FI (OR: 1.27, 95% CI: 1.11–1.46), excellent outcomes (OR: 1.21, 95% CI: 1.03–1.43), SR (OR: 1.23, 95% CI: 1.05–1.45), mortality (OR: 0.72, 95% CI: 0.54–0.97) in crude analyses. In adjusted analyses, the MT + IVT group reduced the risk of mortality (OR: 0.65, 95% CI: 0.49–0.88). However, the difference in FI between the MT + IVT group and the MT alone group was not significant (OR: 1.17, 95% CI: 0.99–1.38, Fig. 3a). For observational studies, the results of FI (OR: 1.34, 95% CI: 1.16–1.33), excellent outcomes (OR: 1.30, 95% CI: 1.09–1.54), SR (OR: 1.23, 95% CI: 1.05–1.44), mortality (OR: 0.70, 95% CI: 0.64–0.77) in the MT + IVT group were better. Additionally, the MT + IVT group increased the risk of hemorrhagic transformation (HT) including sICH (OR: 1.16, 95% CI: 1.11–1.21) and aICH (OR: 1.24, 95% CI: 1.05–1.46) in crude analyses. In adjusted analyses, significant better outcomes were seen in the MT + IVT group on FI (OR: 1.36, 95% CI: 1.21–1.52), excellent outcomes (OR: 1.49, 95% CI: 1.26–1.75), and mortality (OR: 0.73, 95% CI: 0.56–0.94). The MT + IVT therapy did improve the prognosis for AIS patients and did not increase the risk of HT compared with MT alone therapy.

## Introduction

Stroke is the second greatest cause of mortality and the leading causes of disability worldwide. According to the Global Burden of Disease Study 2019, the burden of stroke is steadily rising, especially in low- and middle-income nations^1^. Ischemic and hemorrhagic strokes are the two main subtypes, with ischemic strokes accounting for around 85% of instances^2^. Intravenous thrombolysis (IVT) and mechanical thrombectomy (MT) are routinely performed in acute ischemic stroke (AIS) patients with occlusion of anterior circulation. According to the latest guidelines, the treatment window for MT was expanded up to 16–24 h, and IVT with alteplase was approved for patients within 4.5 hours^3^.

The prognosis of AIS was greatly improved when comparing MT with routine medical care^4^. However, there has been controversy regarding the effectiveness of IVT before MT. Most studies indicated that bridging treatment can encourage successful recanalization (SR)^5–9^. IVT, however, raised potential complications, especially intracranial hemorrhage and distal embolization. IVT-induced thrombus fragmentation would make subsequent MT more difficult^10,11^. These conflicting results highlighted the challenges of clinical operation selection.

Currently, several systematic and meta-analysis have compared the MT alone and bridging treatment (MT + IVT)^12–14^. Katsanos et al. indicated that AIS patients with MT + IVT treatment, compared to MT alone treatment, improved functional independence (FI), SR, and three-month mortality results^12^. In direct contrast, one study showed no statistically significant difference between the two treatment^13^. We also found either an assessment limited to observational studies or just randomized controlled trials (RCTs)^12–14^. Given the increasing number of clinical trials in this field, a comprehensive systematic review and meta-analysis should be conducted once more. The evaluations of therapeutic interventions would fall into two categories, observational studies and RCTs.

## Methods

### Literature search strategy

This study was carried out in compliance with the Preferred Reporting Items for Systematic Reviews and Meta-Analysis statement (PRISMA)^15^. This research has been registered via PROSPERO (CRD42022345385). Two investigators searched from four databases (PubMed, Embase, WOS, and Cochrane Library) published From January 2011 to June 2022. Our search strategy combined Medical Subject Headings (MeSH) and free words.

### Selection criteria

The selection criteria were based on the PICOS (population, intervention, comparison, outcomes, and study design) approach. The following criteria served as the basis for our study screening. Inclusion criteria: (1) The studies were observational studies and RCTs; (2) Data from adults (age ≥ 18); (3) Studies provided the quantitative estimates and their 95% confidence interval (95% CI). Exclusion criteria: (1) Studies were literature reviews, protocols, case reports, comments, editorial articles, cell experiments, or animal experiments; (2) Patients of AIS with non-anterior circulation in large vessel occlusion (LVO).

### Participants and interventions

We included AIS patients with LVO in the anterior circulation. Each participant received the MT alone or IVT + MT therapy. Most of included studies primarily used the medication alteplase. It should be highlighted that we did not exclude some other IVT medications from our analysis even though they were not recommended by the guidelines.

### Outcomes

In this study, FI for three months or hospital discharge, defined as a modified Rankin Scale (mRS) score (range,0 to 2), was selected as the primary efficacy outcome. The primary safety indicator was symptomatic intracerebral hemorrhage (sICH) at 24 or 36 h according to Heidelberg Bleeding Classification (HBC) ^16^, or European Cooperative Acute Stroke Study 3 classification (ECASS III)^17^, or ECASS II, or Safe Implementation of Thrombolysis in Stroke-Monitoring Study (SITS-MOST) criteria^18^.

Thrombolysis in Cerebral Infarction (TICI score of 2B, 2C, or 3), modified TICI (mTICI) score (2B or 3), or eTICI score (2B, 2C, or 3)^19^ was defined as SR with final cerebral angiography, and mRS score (range, 0 to 1) were adopted as secondary efficacy outcomes. Mortality at three months or discharge and any intracerebral hemorrhage (aICH) were analyzed as secondary safety outcomes.

### Quality assessment

Given that we had both RCTs, and observational studies included, we employed the Cochrane Risk of Bias tool (RoB) to assess RCTs, which included blinding, baseline comparison, allocation concealment, and randomization analysis. The modified Newcastle–Ottawa Scales (NOS) were used to assess the authenticity and quality of observational studies^20^. The NOS consisted of three sections: patient selection, study group comparability, and outcome assessment. The methodological quality of studies was assessed using a star system. The NOS can award up to nine points, with NOS ≥ 7 indicating high-quality study. Beyond this, the study was considered “low quality”.

### Sensitivity and publication bias analyses

We performed sensitivity analyses to test the stability of our results by excluding each study one by one. Moreover, contour-enhanced funnel plots, Peter's test and Egger's test were conducted only when at least 10 studies were available to detect publication bias.

### Data extraction

Two investigators reviewed each title, abstract, and full-text articles individually to select eligible studies. Any controversies were addressed in discussions with the third author. A Microsoft Excel file had the extracted data that was present. Study title, authors, publication date, study setting, study design, study period, participants, FI, SR, sICH, and mortality definitions, other important outcomes, and adjustment methods were among the extracted study characteristics. Crude data and effects estimate with their 95% CI of crude and adjusted were also included. For more details or unpublished data from conference abstracts, the corresponding authors would be contacted.

### Data synthesis and statistical analysis

Considering the heterogenicity of the methodology, data source, and so on existed in the included studies. We evaluated the inter-study heterogeneity using *I*^2^ tests and the *P*-value. *I*^2^ values < 25%, 25–50%, 50–75%, 75–100% indicated no, moderate, large, and high levels of heterogeneity, respectively. *P*-value < 0.1 was considerately statistically significant. For RCTs, the Mantel–Haenszel fixed-effects model was used if *I*^2^ < 50%. Otherwise, the random-effects model was applied. For observational studies and subgroups analysis, wo chose the random-effects model to control the potential bias. After thoroughly reviewing each included study, we analyzed crude data and adjusted data separately to increase the credibility. These methods were applicable to both crude data analysis and adjusted analysis. For studies that used covariates, we included data that was adjusted for covariates by the original authors in the adjusted analysis.

Also, we performed subgroup analysis by study design (prospective study and retrospective study), and study area (Asia, European, and America). All the analyses were conducted in the RevMan software version 5.3 and computer program R software version 4.1.1. Unless otherwise noted, all *P*-values were two-tailed and less than 0.05 was considered statistically significant.

### Ethical approval

This article belonged to the category of systematic review and meta-analysis, and we have confirmed that no ethical approval is required.

## Results

### Literature retrieval and study characteristics

**Figure 1:**
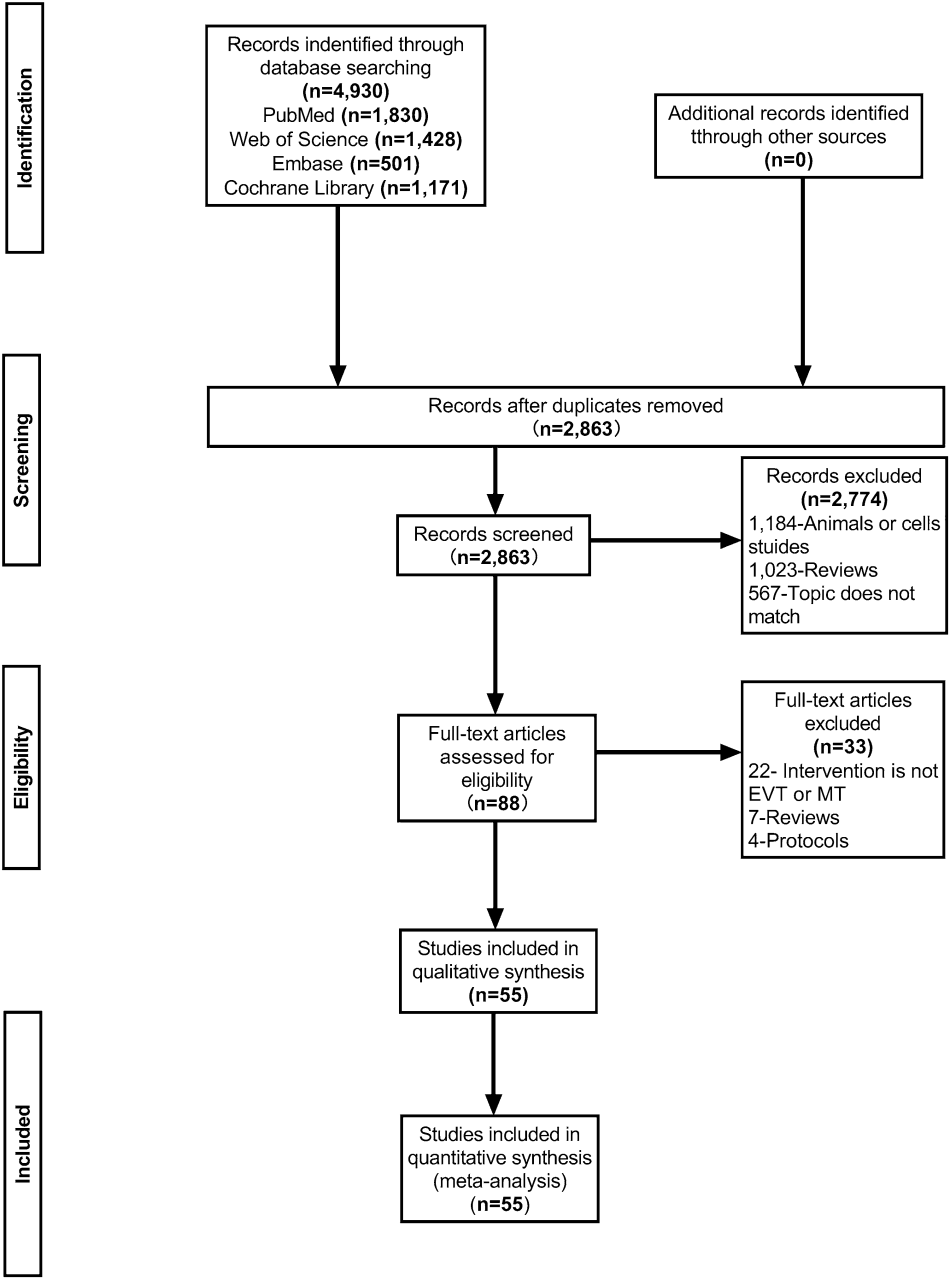
PRISMA flow diagram of literature selection.

The study process as shown in Fig. 1. There were 4,930 items in total (1,830 from PubMed, 1,428 from WOS, 501 from Embase, and 1,171 from Cochrane Library). 2,863 items were included in the abstract screening after eliminating duplicates. Then 2,774 unrelated studies were excluded. Among the 2774 studies, 1184 were basic experimental studies involving animals and cells, 1023 were reviews, and 567 were studies that did not match the research topic. A total of 88 full-text articles were assessed for eligibility. We excluded 33 studies, 22 of which used therapies other than MT or IVT, seven studies were reviews, and four pieces involved RCTs protocol. Finally, 55 studies^6–9,21–70^ met our protocol and were qualitatively synthesized and meta-analyzed.

Table 1 displays the characteristics of eligible studies, including the authors and years of publication, study design type, study period, study participants, age, gender, NIHSS score, location of occlusion, FI definition, SR definition, sICH definition, mortality definition, other outcomes, adjustment method, and adjustment of confounding factors. The study evaluated data from 17 nations, including 10 from Europe, four from Asia, two from The North American, and the one from Australia. Nine RCTs and 46 observational studies—29 retrospective (RS), 16 prospective (PS), and one cross-sectional (CS) were included in the analysis. almost all studies used an mRS score ≤ 2 to define FI. Methods to define SR included TICI 2b/3, mTICI 2b/3, and eTICI 2b/3. Additionally, several methods were adopted to assess sICH (ECASS II/III, HBC, and SITS-MOST). A portion of included studies adopted multivariate analysis, multivariate binary logistic regression, and propensity score method (PSM) to adjust the data.

**Table 1:** Characteristics and treatment strategies of included studies.

Study (Author, year, country)	Study design	Period	Study participants MT + IVT/dMT	Age, mean, y MT + IVT/dMT	Male MT + IVT/dMT	NIHSS, median MT + IVT/dMT	Location of occlusion MT + IVT/dMT	FI definition	SR definition	sICH definition	Mortality definition	Other outcomes	Adjustment method	Adjustment of confounding factors
Coutinho, et al. 2017, Canada^21^	RCT	SWIFT:2010.01–2011.11 STAR: 2010.01–2012.01	291 160/131	67/69	63/58	17/17	Carotid: 31/63 M1: 99/88 M2 or M3: 23/17 Posterior circulation:0/1	mRS 0–2 (90d)	mTICI 2b/3	ECASS II	All cause (90d)	subarachnoid hemorrhage	NA	NA
Gariel et al. 2018, France^9^	RCT	2015.10–2016.10	381 250/131	68.7/72.2	142/65	17/18	M1: 174/96 M2: 39/14 Intracranial ICA: 33/18	mRS 0–2 (90d)	mTICI 2b/3	ECASS III	All cause (90d)	the change in NIHSS score at 24 h	NA	age, hypertension, diabetes mellitus, admission NIHSS, and ASPECTS site of occlusion, onset to puncture time
Chalos et al. 2019, Dutch^22^	RCT	2014,03–2016.06	1485 1161/324	70/72	621/172	16/17	ICA: 70/12 ICA-T: 241/1101 M1: 637/186 M2: 142/33	mRS 0–2 (90d)	eTICI 2b/2C	HBC	All cause (90d)	NA	logistic regression	Age, baseline NIHSS, history of diabetes mellitus; pre-stroke mRS, prior use of anticoagulant medication, onset to-first noncontract-computed-tomography time
Yang et al. 2020, China^23^	RCT	2018.02–2019.07	656 329/327	69/69	181/189	NA	Intracranial ICA: 114/112 M1: 178/161 M2: 326/320	mRS 0–2 (90d)	eTICI score ≥ 2b	HBC	All cause (90d)	EQ-5D-5L score at 90 days	logistic regression	NA
LeCouffe et al. 2021, Europe^24^	RCT	2018.01–2020.10	539 266/273	69/72	144/161	16/16	Intracranial ICA: 0/4 Terminal ICA: 50/64 M1: 174/156 M2: 40/45	mRS 0–2 (90d)	mTICI 2b/3	HBC	All cause (90d)	EQ-5D-5L score at 90 days	logistic regression	age, baseline NIHSS score, collateral status, prestroke score on the mRS, and time from symptom onset to randomization
Zi et al. 2021, China^25^	RCT	2018.05–2020.05	234 118/116	70/70	66/66	16/16	Intracranial ICA: 17/18 M1: 99/95	mRS 0–2 (90d)	eTICI 2b/2C/3	ECASS III	All cause (90d)	EQ-5D-5L score at 90 days	logistic, ordinal logistic, or linear regression model	age, baseline NIHSS score, baseline ASPECTS, onset to randomization time, and occlusion site
Suzuki et al. 2021, Japan^26^	RCT	2017.01–2019.01	204 103/101	76/74	72/56	17/19	ICA: 36/41 M1 proximal: 18/17 M1 distal: 49/41	mRS 0–2 (90d)	eTICI 2b/2C/3	SITS-MOST	All cause (90d)	NA	logistic regression	NA
Huu An et al. 2022, Vietnam^27^	RCT	2020.11–2021.11	60 30/30	66.5/64	21/18	13/12	ICA: 12/10 M1: 15/18 M1: 3/2	mRS 0–2 (90d)	TICI 2b/3	NA	All cause (90d)	NA	NA	NA
Sakai et al. 2022, Japan^28^	RCT	2017.06–2020.01	41 32/9	74.5	18/4	20	ICA: 10/3 M1: 22/6	mRS 0–2 (90d)	TIMI	ECASS	NA	NA	NA	NA
Dávalos et al. 2012, France^29^	RS	2010.06––	147 74/67	66.2/66.4	29/33	17/18	Cervical ICA: 1/5 Terminus ICA: 20/13 M1: 34/32 M2: 12/7	mRS 0–2 (90d)	mTICI 2b/3	SITS-MOST	All cause at 90d	Neurological outcome was measured by NIHSS score	NA	NA
Pfefferkorn et al. 2012, Germany^30^	RS	2003.01–2010.06	49 26/23	62.1/64.6	16/9	19.0/19.6	M1: 6/9 CT: 2/2 BA: 18/12	mRS 0–2 (90d)	TIMI II/III	NA	All cause (90d)	NA	NA	NA
Kass-Hout et al. 2014, America^31^	RS	2005–2010	104 42/62	NA	9/40	NA	MCA	mRS 0–2 (90d)	TICI 2b/3	ECASS III	All cause at discharge	NIHSS score at discharge	NA	NA
Leker et al. 2015, Israel^32^	RS	2010.12–2014.10	57 24/33	66.8/64.4	8/15	19.5/20	Cardioembolic: 16/25 LVO: 5/4 Other: 1/0 Unknown: 2/0	mRS 0–2 (90d)	TICI 2b/3	NA	NA	NA	Multivariate analysis	admission NIHSS, age, the presence of symptomatic hemorrhage
Maier et al. 2015, Germany^33^	PS	2014.01–2015.11	109 81/28	75/76	40/12	17/12.5	NA	mRS 0–2 (90d)	TICI 2b/3	ICH within 48 h or ≥ 4 NIHSS points increase	All cause (90d)	Change in NIHSS	NA	NA
Guedin et al. 2015, France^34^	RS	2011.01–2013.06	68 28/40	69.2/64.6	11/15	18/15	NA	mRS 0–2 (90d)	TICI 2b/3	SITS-MOST	All cause at 90d	NA	NA	NA
Broeg-Morvay et al. 2016, Switzerland^35^	RS	2009.02–2014.08	196 156/40	73/77	82/25	15/17	ICA: 64/21 M1: 83/16 M2: 9/3	mRS 0–2 (90d)	TICI 2b/3	NA	All cause (90d)	NA	PSM	NA
Behme et al. 2016, Germany^36^	RS	2012.07–2013.12	93 66/27	74/74	34/9	16/17	MCA	NA	mTICI 2b/3	NA	NA	NIHSS score at discharge of 10 points or higher	NA	NA
Minnerup et al. 2016, Germany^70^	PS	2012.04–2013.08	1107 504/1543	68.3/68.7	304/122	15/7	ICA: 198/174 M1: 268/188 M2: 62/67 Posterior circulation: 65/68 Other: 4/6 Undetermined: 6/1	mRS 0–2 (90d)	NA	NA	All cause (90d)	NA	PSM	NA
Abilleira et al. 2017, Spain^37^	RS	2011.01–2015.10	1166 567/599	68.6/68.1	306/309	17/17	ICA: 194/204 MCA: 372/394	mRS 0–2 (90d)	mTICI 2b/3	NA	All cause at 90d	NA	PSM	NA
Bellwald et al. 2017, Switzerland^38^	PS	Essen: 2012.06–2013.08 Bern: 2009.01–2014.08	360 249/111	73/75	127/61	16/15	ICA: 102/53 M1: 122/43 M2: 25/15	mRS 0–2 (90d)	TICI 2b/3	ECASS III	All cause at 90d	NA	PSM	age, sex, NIHSS, time from symptom onset to diagnosis, hypertension, and thrombus location (ICA or MCA)
Weber, et al. 2017, Germany^39^	RS	2012.06–2013.08	250 105/145	70.2/69.3	57/67	15.5/15	ICA: 19/30 Carotid T: 21/20 M1: 48/52 M2: 20/23 BA: 10/21 Other: 6/4	mRS 0–2 (90d)	TICI 2b/3	ECASS III	All cause at 1 year	NA	multivariate binary logistic regression	age, NIHSS score at admission, arterial hypertension, diabetes mellitus, current smoking, atrial fibrillation, occlusion of the internal carotid artery, time from symptom onset to first cerebral imaging, time from symptom onset to end of the thrombectomy procedure, SR
Alonso de Leciñana, et al. 2017, Spain^40^	PS	NA	71 53/21	74/64	9/24	17/19	ICA-T: 13/4 M1: 30/10 M2: 2/2 BA: 3/3 Tandem: 5/2	mRS 0–2 (90d)	TICI 2b/3	SITS-MOST	All cause at 90d	NA	multivariate logistic regression	NA
Froehler et al. 2017, America^41^	PS	2014.08–2016.06	905 579/326	67.8	533	NA	ICA: 223 M1: 541 M2: 172 Other: 48	mRS 0–2 (90d)	mTICI 2b/3	NA	All cause at 90d	NA	PSM	age, NIHSS and time from onset were regarded as continuous predictors while occlusion location, tPA and transfer status, Time from onset, as the continuous predictor of principal interest
Rai et al. 2017, America^42^	RS	NA	90 38/52	63/69	20/20	18/16	ICA-T: 4/8 MCA: 34/44	mRS 0–2 (90d)	TICI 2b/3	ECASS-II	All cause (90d)	Home discharge	NA	NA
Wang et al. 2017, China^43^	RS	2014.01–2016	276 138/138	67/67	78/76	17/16	ICA-P: 16/13 ICA-T: 33/46 M1: 86/69 M2: 6/10	mRS 0–2 (90d)	mTICI 2b/3	HBC	All cause (90d)	NA	PSM	age, sex, previous stroke, pre-morbid mRS, time from onset to door, stroke etiology, occlusion site, baseline ASPECTS, baseline NIHSS score, collateral status
Wee et al. 2017, Australia^44^	RS	2013.10–2016.04	50 21/29	73/71	8/16	15/15	ICA: 5/6 M1: 11/12 M2: 5/11 TAO: 4/3	NA	mTICI 2b/3	ECASS III	NA	improvement in NIHSS score at 24 h	NA	NA
Merlino et al. 2017, Italy^45^	PS	2015.01–2016.03	66 33/33	69.6/70.8	15/19	17.5/20	LVO: 4/3 Cardioembolism: 17/19 Other: 10/11	mRS 0–2 (90d)	TICI 2b/3	ECASS III	All cause at 90d	NA	logistic regression	age, NIHSS at admission, pre-stroke mRS, use of anticoagulants at admission, and time from symptoms onset to EVT
Park et al. 2017, Korea^46^	PS	2008–2013	639 458/181	68/69	260/103	15/15	ICA: 183/71 MCA: 226/84 Others: 49/26	mRS 0–2 (90d)	mTICI 2b/3	ECASS III	All cause (90d)	NA	NA	NA
Ferrigno et al. 2018, France^8^	PS	2012.01–2017.01	485 348/137	66.3/67.1	160/61	16.2/16.1	LVO: 59/11 Cardioembolism: 129/83 Other: 160/43	mRS 0–2 (90d)	mTICI 2b/3	ECASS II	All cause at 90d	mRS 0–1 (90d)	multivariable logistic regression	age, hypertension, diabetes mellitus, hypercholesterolemia, smoking, previous or current atrial fibrillation, previous transient ischemic attack or stroke, previous antithrombotic medication, unknown onset stroke, admission NIHSS and ASPECTS scores, onset to puncture time, and site of occlusion
Choi et al. 2018, Korea^47^	PS	2009.01–2017.06	81 43/38	68.9/72.6	29/17	13/15	ICA: 8/9 Tandem: 4/7 Cardioembolic: 26/25 Atherothrombotic: 9/3 M1: 21/17 M2: 9/5	mRS 0–2 (90d)	TICI 2b/3	4 points or more on the NIHSS score	All cause at 90d	NA	multivariate logistic regression	NA
Al-Khaled et al. 2018, Germany^48^	RS	2011.07–2016.12	236 144/92	69/68.7	62/46	13/13	NA	mRS 0–2 (90d)	mTICI 2b/3	NA	All cause at 90d	NA	logistic regression	NA
Heinrichs et al. 2018, Germany^49^	PS	2015.01–2016.03	188 118/70	74.1/73.4	59/29	17/18	Cardioembolic: 72/39 Large artery: 25/19 Other: 21/12	mRS 0–2 (90d)	TICI 2b/3	ECASS II	All cause (90d)	NA		NA
Casetta et al. 2019, Italy^7^	RS	2011–2015	1,148 635/513	68.3/68.5	NA	18/18	NA	mRS 0–2 (90d)	TICI 2b/3	ECASS II	All cause (90d)	NA	PSM	age, sex, history of hypertension, diabetes, prior stroke or transient ischemic attack, atrial fibrillation, use of oral anticoagulants, known carotid stenosis > 70%, wakeup stroke, time interval from symptom onset to hospital arrival, site of arterial occlusion
Di Maria et al. 2018, France^6^	PS	2012.11–2016.11	1507 972/531	66.9/66.1	530/272	17/16	M1: 499/169 M2: 129/65 Carotid T: 168/113 Tandem: 180/84	mRS 0–2 (90d)	mTICI 2b/3	ECASS II	All cause (90d)	NA	PSM	NA
Sallustio et al. 2018, Italy^50^	RS	2009.08–2017.06	325 193/132	71.8/70.3	111/74	19/19	MCA: 122/79 ICA-T: 4/1 Tandem: 62/46 Other: 5/6:	mRS 0–2 (90d)	TICI 2b/3	ECASS III	All cause (90d)	NA	logistic regression analysis	age, NIHSS
Bourcier et al. 2018, France^51^	RS	2014.01–2016.06	141 85/56	68/73	55/24	18/18	NA	mRS 0–2 (90d)	mTICI 2b/3	NA	All cause at 90d		PSM	age, gender, wake-up stroke, hypertension, dyslipidemia, smoking, diabetes, obesity, imaging at the NIC, baseline NIHSS score, time from onset to imaging, baseline ASPECTS, cervical lesion, anterior collaterality
Goyal et al. 2018, America^52^	RS	2013.05–2015.05	569 292/277	62.5/61.0	139/160	17/16	M1:156/133 M2: 37/27 M3: 1/1 ACA: 2/2 TICA: 32/27 ICA + MCA: 40/39 VA/BA: 24/5	mRS 0–2 (90d)	mTICI 2b/3	SITS-MOST	All cause at 90d	NA	PSM	age, gender, admission NIHSS score, posterior circulation, onset to groin puncture, IVT pretreatment
Leker et al. 2018, Israel^53^	RS	2014.01–2016.03	270 156/111	68.1/67.4	91/58	16/16	Cardioembolic: 75/62 LVO: 32/17 Other: 52/32	mRS 0–1 (90d)	TICI 2b/3	NA	All cause at discharge	NIHSS score – day 1 < 2; hospital discharge	multivariate regression analysis	admission NIHSS score, age, gender, time to endovascular treatment, stroke subtype
Guimarães Rocha et al. 2019, Portugal^54^	RS	2015.01–2017.06	234 152/82	70.9/71.93	40/39	16.28/15.67	ICA-T: 18/8 Intracranial ICA: 48/16 M1: 77/50 M2: 27/16	mRS 0–2 (90d)	mTICI 2b/3	ECASS III	All cause at 90d	NA	multivariate logistic regression	age, NIHSS score, ASPETS, intracranial ICA occlusion, cardioembolic stroke, and time from symptom onset to recanalization
Balodis et al. 2019, Germany^55^	PS	2014.02–2017.01	146 84/62	72/72	38/28	15/16.5	ICA or M1: 17/19	mRS 0–2 (90d)	TICI 2b/3	large parenchymal hematoma (> 30% blood of stroke volume with mass effect and increase of 4 points or more in the NIHSS score)	All cause at 90d	NA	logistic regression	NA
Gong et al. 2019, China^56^	RS	2015.11–2018.01	42 21/21	69/71	27/15	13/15	MCA	mRS 0–2 (90d)	TICI 2b/3	NA	All cause at 90d	NA	Multivariate Matching	NA
Goyal et al. 2019, America^52^	RS	2013.01–2017.11	419 287/132	63.7/64.3	143/66	16/16	M1: 121/40 M2: 31/12 M3/M4: 7/ ICA: 30/14 M1 + ICA: 30/17 M3/M4:1/7 Posterior: 24/36	mRS 0–2 (90d)	mTICI 2b/3	SITS-MOST	All cause at 90d	Discharge NIHSS	Multivariable logistic regression analyses	age, race, gender hyperlipidemia, atrial fibrillation, coronary artery disease, hypertension, smoking, diabetes, congestive heart failure, prior stroke, intravenous thrombolysis, pretreatment with antiplatelets, pretreatment with oral anticoagulant, pretreatment with statin, SBP at admission, DBP at admission, NIHSS score at admission, ASPECTS at baseline, good collaterals, symptom onset-to-groin puncture time, admission blood glucose, occlusion site in anterior circulation
Maingard et al. 2019, Ireland^57^	RS	2010.06–2016.06	355 210/145	66/68	116/81	17/17	NA	mRS 0–2 (90d)	mTICI 2b/3	NA	All cause at 90d	NA	Multivariable regression	age, sex, hypertension, hyperlipidemia, ischemic heart disease, diabetes, atrial fibrillation, previous stroke, NIHSS, ASPECTS, collateral grade, general an aesthesia
Hassan et al. 2019, America^58^	RS	2012.01–2018.08	254 96/158	66/68	116/81	17/17	NA	mRS 0–2 (90d)	mTICI 2b/3	≥ 4 point NIHSS score worsening within 24 h	All cause at 90d	Hospital encounter charges; Hospital final bill	NA	age, sex, hypertension, hyperlipidemia, ischemic heart disease, diabetes, atrial fibrillation, previous stroke, NIHSS, ASPECTS, collateral grade, general an aesthesia
Reiff et al. 2020, Germany^59^	RS	2009–2019	168 44/124	76	79	16	ICA: 3/4 ICA + M1: 10/15 Carotid T: 11/25 M1: 18/54 M2: 2/26	mRS 0–2 (90d)	mTICI 2b/3	ECASS II	NA	NA	multivariate logistic regression	NIHSS score on admission, mTICI score, symptom onset to treatment time
Yi et al. 2020, Korea^60^	RS	2015.01–2019.01	177 123/177	71.8/70.1	65/97	NA	ACA: 5/5 M1: 53/73 M2: 12/10 Proximal ICA: 33/58 Posterior circulation: 11/11	mRS 0–2 (90d)	TICI 2b/3	CT scan	All cause at 90d	NA	NA	NA
Hinsenveld et al. 2020, Netherlands^61^	RS	2014.03–2017.11	1427 1023/404	71/73	557/189	16/15	NA	NA	eTICI 2b/3	HBC	NA	mRS score at discharge	NA	age, sex, National Institutes of Health Stroke Scale score, baseline modified Rankin Scale score, systolic blood pressure, anticoagulation (coumarins or direct oral anticoagulants), periprocedural local anesthesia only
Imbarrato et al. 2020, America^62^	RS	2011–2015	46 23/23	69.2/71.1			M1: 10/11 M2: 5/7	mRS 0–2 (90d)	TICI 2b/3	NA	NA	NA	NA	NA
Jian et al. 2021, China^63^	PS	2017.11–2019.03	482 187/295	72/73	87/172	17/18	LVO: 75/123 Cardioembolism: 89/128 Other: 22/43	mRS 0–2 (90d)	mTICI ≥ 2b	HBC	All cause at 90d	NA	multivariate logistic regression	age, preoperative anticoagulants, aspiration catheter, intraoperative heparin, and the use of IVT, baseline NIHSS, ASPECTS, location of the occluded vessels, TOAST classification
Tong et al. 2021, China^64^	PS	2017.11–2019.03	973 405/568	64/66	266/392	16/17	ICA: 111/169 M1: 192/239 M2: 40/54 Vertebrobasilar artery: 75/120 Other: 114/137	mRS 0–2 (90d)	mTICI 2b/3	HBC	All cause at 90d	intraprocedural embolization	PSM	age, sex, NIHSS score, history of dyslipidemia, prior ischemic stroke, admission mode, prior use of antiplatelet agents, and prior use of anticoagulants
Kandregula et al. 2021, America^65^	RS	2017–2018	2,895 1,226/1,669	NA	NA	NA	NA	mRS 0–2 (90d)	NA	NA	NA	the presence of ICH, IVH, SAH, vasospasm, and access-site hemorrhages	NA	NA
Zha et al. 2021, China^66^	PS	2018.03–2019.07	130 65/65	68/68	37/37	15/16	ICA: 16/23 Posterior circulation: 6/4	mRS 0–2 (90d)	mTICI 2b/3	ECASS II	All cause at 90d	NA	PSM	NA
Machado et al. 2021, Portugal^67^	RS	2016.01–2018.11	524 347/177	73/75	158/89	17/16	NA	mRS 0–2 (90d)	mTICI 2b/3	ECASS II	All cause at 90d	NA	NA	NA
Platko et al. 2022, America^68^	RS	2017.01–2019.12	172 82/89	70/71	39/27	18/15	NA	mRS 0–2 (90d)	TICI 2b/3	NA	All cause at 90d	NA	NA	NA
Dicpinigaitis et al. 2022, America^69^	CS	2015–2018	48,525 28,790/19,735	68.9/69.7	9,685/13,535	NA	NA	NA	NA	NA	NA	NA	Multivariable logistic regression	NA

*AIS* Acute ischemic stroke, *ACA* Anterior cerebral artery, *ASPECTS* Alberta stroke program early CT score, *ASITN/SIR* American society of interventional and therapeutic neuroradiology/Society of interventional radiology, *aICH* Any intracerebral hemorrhage, *BA* Basilar artery, *CT* Carotid terminus, *CS* Cross-sectional study, *CI* Confidence interval, *DBP* Diastolic blood pressure, *dMT* Direct mechanical thrombectomy, *SBP* Systolic blood pressure, *EQ-5D-5L* The EuroQoL group 5-dimension 5-level self-report questionnaire, *ECASS II* European cooperative acute stroke study 2 classification, *ECASS III* European cooperative acute stroke study 3 classification, *EVT* The endovascular treatment, *FI* Functional independence, *HBC* Heidelberg bleeding classification, *IVT* Intravenous thrombolysis, *ICA-T* Internal carotid artery terminus, *ICA-P* Proximal ICA, *LVO* Large vessel occlusion, *MT* Mechanical thrombectomy, *MCA* Middle cerebral artery, *M1* M1 segment of the middle cerebral artery, *M2* M2 segment of the middle cerebral artery, *mRS* modified Rankin Scale, *mTICI* modified Thrombolysis in Cerebral Infarction, *NIHSS* national institutes of health stroke scale, *NOS* Newcastle–Ottawa scales, *PSM* Propensity score matching, *PS* Prospective study, *RS* Retrospective study, *SITS-MOST* Safe implementation of thrombolysis in stroke-monitoring study, *SR* Successful recanalization, *sICH* Symptomatic intracerebral hemorrhage.

### Quality assessment for included studies

According to RoB, most trials were of high quality and possessed a low overall risk of bias. Supplemental Fig. 11 showed the specific details. Due to randomization and blinding items, a trial had a high risk of bias^27^. Additionally, Supplemental Table 4 showed how detailed information from OS were evaluated using the NOS scale. Except for one study^62^, which scored only 6 because controls for comparability between the two groups were omitted from the study. All other studies were rated as “high quality”.

### Crude analysis

#### Primary outcomes

**Figure 2:**
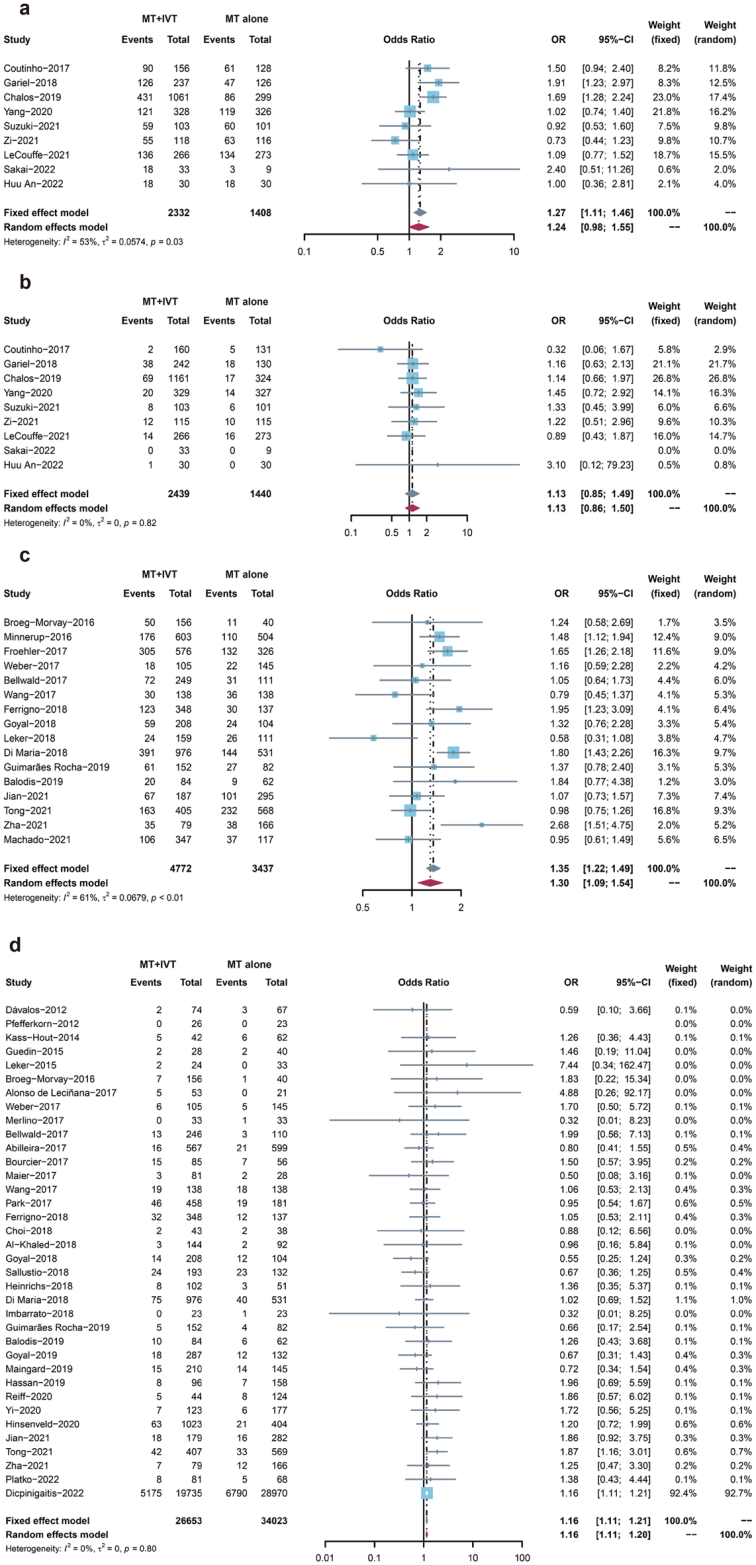
the forest plot of primary outcomes of crude data. (**a**) FI of RCTs. (**b**) sICH of RCTs. (**c**) FI of OS. (**d**) sICH of observational studies.

The results would be reported separately by RCTs and observational studies. Regarding efficacy outcomes, data from the nine RCTs indicated that MT + IVT group had superior FI than the MT alone group (OR: 1.27, 95% CI: 1.11–1.46, Fig. 2a), with large heterogeneity (*I*^2^ = 53%, *P* = 0.03). About safety outcomes, the results of sICH showed no significant difference between the two groups (OR: 1.13, 95% CI: 0.86–1.49, Fig. 2b), indicating no heterogeneity (*I*^2^ = 0, *P* = 0.82). Overall, 40 observational studies reported the results for FI, suggesting better results were seen in the MT + IVT group (OR: 1.34, 95% CI: 1.16–1.33, Fig. 2c), with large heterogeneity (*I*^2^ = 70%, *P* < 0.01). Data on sICH was extracted from 36 observational studies and found a 16% higher risk of HT (OR: 1.16, 95% CI: 1.11–1.21, Fig. 2d) in the MT + IVT group, with no heterogeneity (*I*^2^ = 0, *P* = 0.80).

#### Secondary outcomes

**Figure 3:**
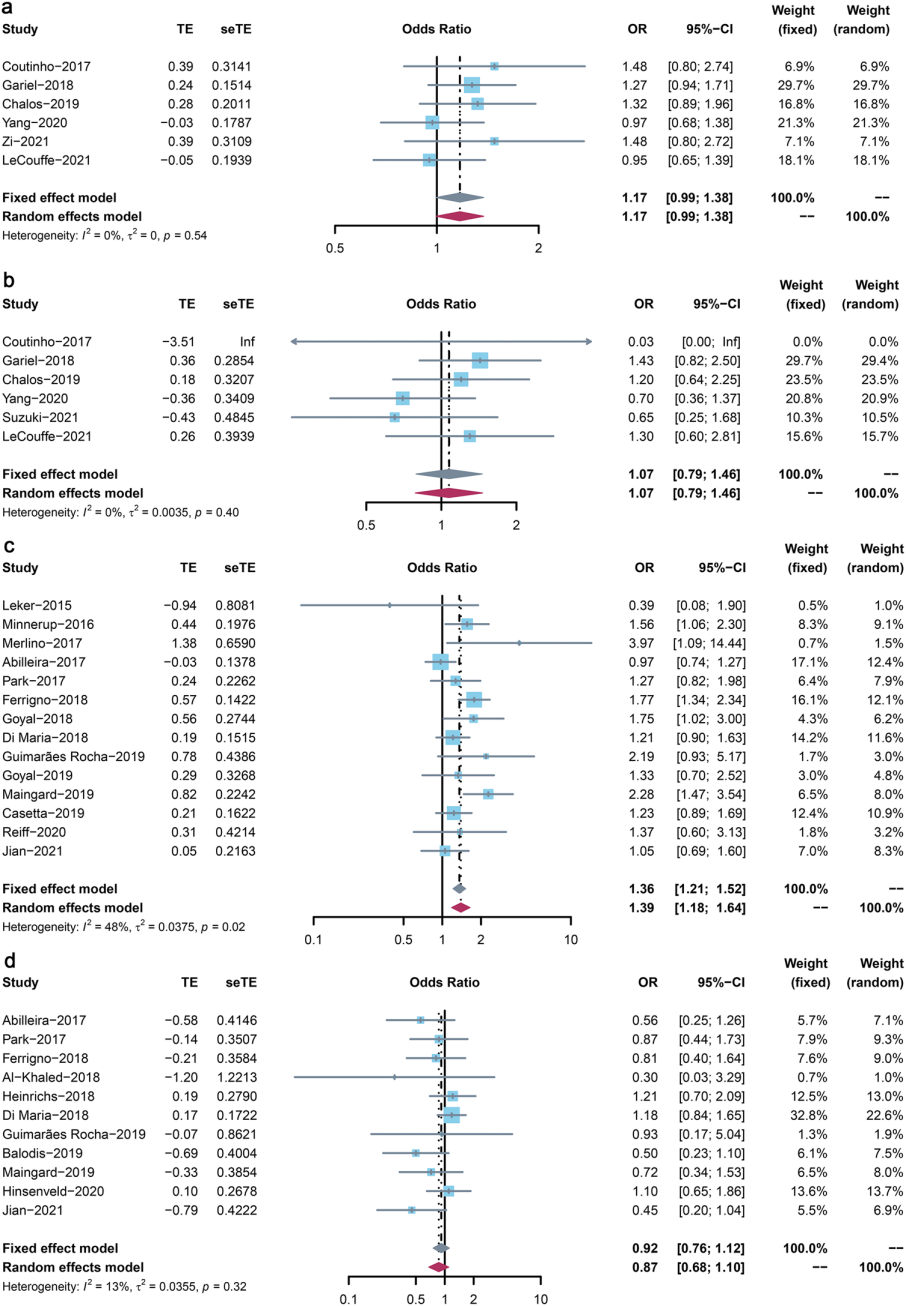
the forest plot of primary outcomes of adjusted data. (**a**) FI of RCTs. (**b**) sICH of RCTs. (**c**) FI of OS. (**d**) sICH of observational studies.

On the secondary efficacy outcomes, in nine RCTs, the MT + IVT group outperformed the MT alone group for excellent outcomes (mRS score: 0–1) (OR: 1.21, 95% CI: 1.03–1.43, Fig. 3a) with moderate heterogeneity (*I*^2^ = 43%, *P* = 0.09). Additionally, the MT + IVT group saw 23% more SR than the MT alone group (OR: 1.23, 95% CI: 1.05–1.45, Fig. 3b) in eight RCTs, no heterogeneity accompanied (*I*^2^ = 0, *P* = 0.96). Regarding safety outcomes of aICH from seven RCTs, the MT + IVT group had a 25% higher risk of HT than the MT alone group (OR: 1.25, 95% CI: 1.00–57, Fig. 3c), with low heterogeneity (*I*^2^ = 22%, *P* = 0.26). Mortality at 3-months or hospital discharge from eight RCTs in the MT + IVT group showed a lower mortality compared to the MT alone group (OR: 0.72, 95% CI: 0.54–0.97, Fig. 3d), with large heterogeneity (*I*^2^ = 54%, *P* = 0.03).

**Figure 4:**
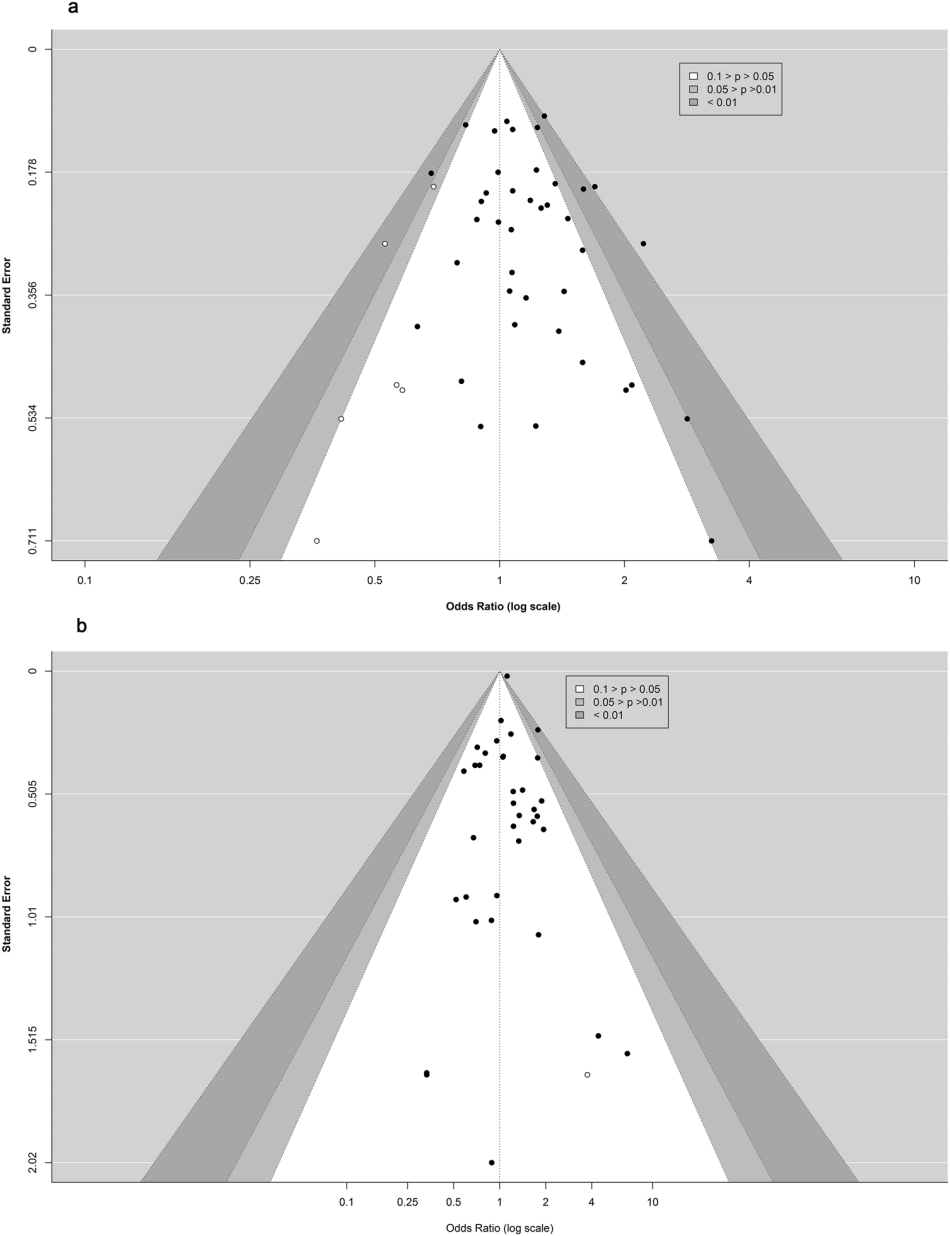
the funnel plot of primary outcomes of crude data about observational studies. (**a**) FI. (**b**) sICH.

For efficacy outcomes, a total of 16 OS reported the excellent outcomes (mRS score: 0–1). Better results were seen in the MT + IVT group (OR: 1.30, 95% CI: 1.09–1.54, Fig. 4a) with large heterogeneity (*I*^2^ = 61%, *P* < 0.01). 38 OS showed SR outcomes, with the MT + IVT group increased the rate of SR (OR: 1.23, 95% CI: 1.05–1.44, Supplemental Fig. 4b), with large heterogeneity (*I*^2^ = 60%, *P* < 0.01). For safety outcomes, the MT + IVT group had higher aICH by 19% than the MT alone group (OR: 1.24, 95% CI: 1.05–1.46, Supplemental Fig. 4c) in 23 observational studies with moderate heterogeneity (*I*^2^ = 44%, *P* = 0.01). Additionally, in 34 investigations, mortality was 30% lower in the MT + IVT group compared to the MT alone group (OR: 0.70, 95% CI: 0.64–0.77, Supplemental Fig. 4d), with moderate heterogeneity (*I*^2^ = 42%, *P* = 0.01).

#### Subgroup analysis

Given the large heterogeneity of some outcomes, subgroup analysis by study design (RS vs PS) and area (Asia vs Europe vs America) was conducted. Regarding subgroup outcomes by study region in the RCTs, there was significant difference between Europe and Asia group in terms of FI (*P* = 0.05), Specifically, the MT + IVT group had better outcomes in Europe (OR: 1.46, 95% CI: 1.07–1.98), whereas there were no significant differences in Asia subgroup between the MT + IVT and the MT alone therapy (OR: 0.95, 95% CI: 0.75–1.21). Moreover, stratifying studies according to mortality showed significant differences (*P* < 0.01,). In Europe, the MT + IVT group reduced mortality risk by 45% (OR: 0.55, 95% CI: 0.45–0.68), while in Asia there was no significant difference (OR: 1.07, 95% CI: 0.78–1.48). There were no significant differences regarding SR (*P* = 0.73), excellent outcomes (*P* = 0.14), sICH (*P* = 0.25), and aICH (*P* = 0.10). The above details were depicted in Supplemental Table 1. On the basis of the results of study area subgroup in OS, no statistically significant variations regarding FI (*P* = 0.28), excellent outcomes (*P* = 0.31), SR (*P* = 0.93), sICH (*P* = 0.63), aICH (*P* = 0.19), and mortality (*P* = 0.38), of which were detailed in Supplemental Table 2.

The results of the subgroup analysis for observational studies were described in more detail below. As shown in Supplemental Table 3, there was no difference in the outcomes of FI (*P* = 0.13), excellent outcomes (*P* = 0.14), SR (*P* = 0.37), sICH (*P* = 0.20), aICH *(P* = 0.70), and mortality (*P* = 0.92).

### Adjusted analysis

#### Primary outcomes

Results by assessing the adjusted ORs among RCTs between the MT + IVT group and the MT alone group were non-significant for both FI (OR: 1.17, 95% CI: 0.99–1.38, Fig. 3a) and sICH (OR: 1.07, 95% CI: 0.79–1.46, Fig. 3b), suggested no heterogeneity (*I*^2^ = 0, *P* = 0.54), and (*I*^2^ = 0, *P* = 0.40), respectively. However, significant better outcomes were seen in the MT + IVT group on FI in observational studies (OR: 1.36, 95% CI: 1.21–1.52, Fig. 3c), with moderate heterogeneity (*I*^2^ = 48%, *P* = 0.02). We did not see the significant differences on sICH (OR: 0.92, 95% CI: 0.76–1.12, Fig. 3d) between groups with low heterogeneity (*I*^2^ = 13%, *P* = 0.32).

#### Secondary outcomes

Results from RCTs indicated that the MT + IVT group significantly decreased the risk of mortality by 35% (OR: 0.65, 95% CI: 0.49–0.88, Supplemental Fig. 3d), with large heterogeneity (*I*^2^ = 52%, *P* = 0.07). All other results were non-significant differences between the two groups regarding excellent outcomes (OR: 1.11, 95% CI: 0.90–1.38, Supplemental Fig. 3a), SR (OR: 0.92, 95% CI: 0.75–1.13, Fig. 3b), and aICH (OR: 0.93, 95% CI: 0.75–1.15, Fig. 3c). The heterogeneities of above analyses were none (*I*^2^ = 0, *P* = 0.89), low (*I*^2^ = 24%, *P* = 0.24), and moderate (*I*^2^ = 63%, *P* = 0.04).

About observational studies, better results were seen in the MT + IVT group about the outcomes of excellent outcomes (OR: 1.49, 95% CI: 1.26–1.75, Supplemental Fig. 4a) with low heterogeneity (*I*^2^ = 4%, *P* = 0.40). We also observed the MT + IVT group reduced the risks of mortality by 27% (OR: 0.73, 95% CI: 0.56–0.94, Supplemental Fig. 4d) with large heterogeneity (*I*^2^ = 67%, *P* = 0.40) between two groups. And no significant differences were seen in the outcomes of SR (OR: 1.21, 95% CI: 0.85–1.74, Supplemental Fig. 4b) with large heterogeneity (*I*^2^ = 74%, *P* < 0.01), and aICH (OR: 1.06, 95%CI: 0.83–1.35, Supplemental Fig. 4c) by large heterogeneity (*I*^2^ = 28%, *P* = 0.22).

#### Subgroup analysis

Due to the limited number of included RCTs, advanced subgroup analysis was performed solely in observational studies. Among the subgroup of study area, there were no distinguishable differences in the outcomes of FI (*P* = 0.25), excellent outcomes (*P* = 0.20), sICH (*P* = 0.31), and mortality (*P* = 0.53), except for SR (*P* = 0.04). Specifically, there was non-significance in Asia between two groups (OR: 0.59, 95% CI: 0.29–1.21). However, in contrast to the MT alone therapy, the MT + IVT therapy raised the rate of SR by 51% in Europe (OR: 1.51, 95% CI: 1.23–1.86). All details were depicted in Supplemental Table 1.

No discernible differences were observable in outcomes of FI (*P* = 0.93), excellent outcomes (*P* = 0.22), SR (*P* = 0.57), sICH (*P* = 0.82) and aICH (*P* = 0.96) within the subgroup of study design between the two groups, except for mortality (*P* = 0.03). In prospective studies, MT + IVT therapy reduced the risk of mortality by 47% (OR: 0.53, 95% CI: 0.43–0.78). Retrospective analyses, however, did not reveal significant differences in the findings (OR: 0.95, 95% CI: 0.68–1.34). All details were displayed in Supplemental Table 3.

#### Sensitivity analysis

The sensitivity analysis of RCTs in crude data showed the effects of sICH (Supplemental Fig. 5b), SR (Supplemental Fig. 5d), and mortality (Supplemental Fig. 5f) were not substantially modified by exclusion of a certain study. The effect size of FI varied (OR: 1.15, 95% CI: 0.97–1.35, Supplemental Fig. 5a) when one study was excluded^22^. When the trial was eliminated^9^, the total effect sizes showed no discernible improvement (OR: 1.18, 95% CI: 0.99–1.40) in the excellent outcome of MT + IVT therapy. When this study was excluded^22^, a similar outcome (OR: 1.07, 95% CI: 0.89–1.28) was observed. And the MT + IVT group did not increase the risk of aICH (Supplemental Fig. 5d) while removing the study^26^ and the trial^25^, the effect sizes were (OR: 1.16, 95% CI: 0.95–1.41) and (OR: 1.18, 95% CI: 0.98–1.44,), respectively. Similar outcomes were seen in the outcome of excellent outcomes (Supplemental Fig. 5c). As followed by the sensitivity analysis of RCTs in adjusted data, the direction of effect size did not change in the outcomes of our interest (Supplemental Fig. 6b–f) except for the FI. The MT + IVT therapy significantly increased FI (OR: 1.23, 95% CI: 1.03–1.48, Supplemental Fig. 6a) after eliminating the study^23^.

**Figure 5:**
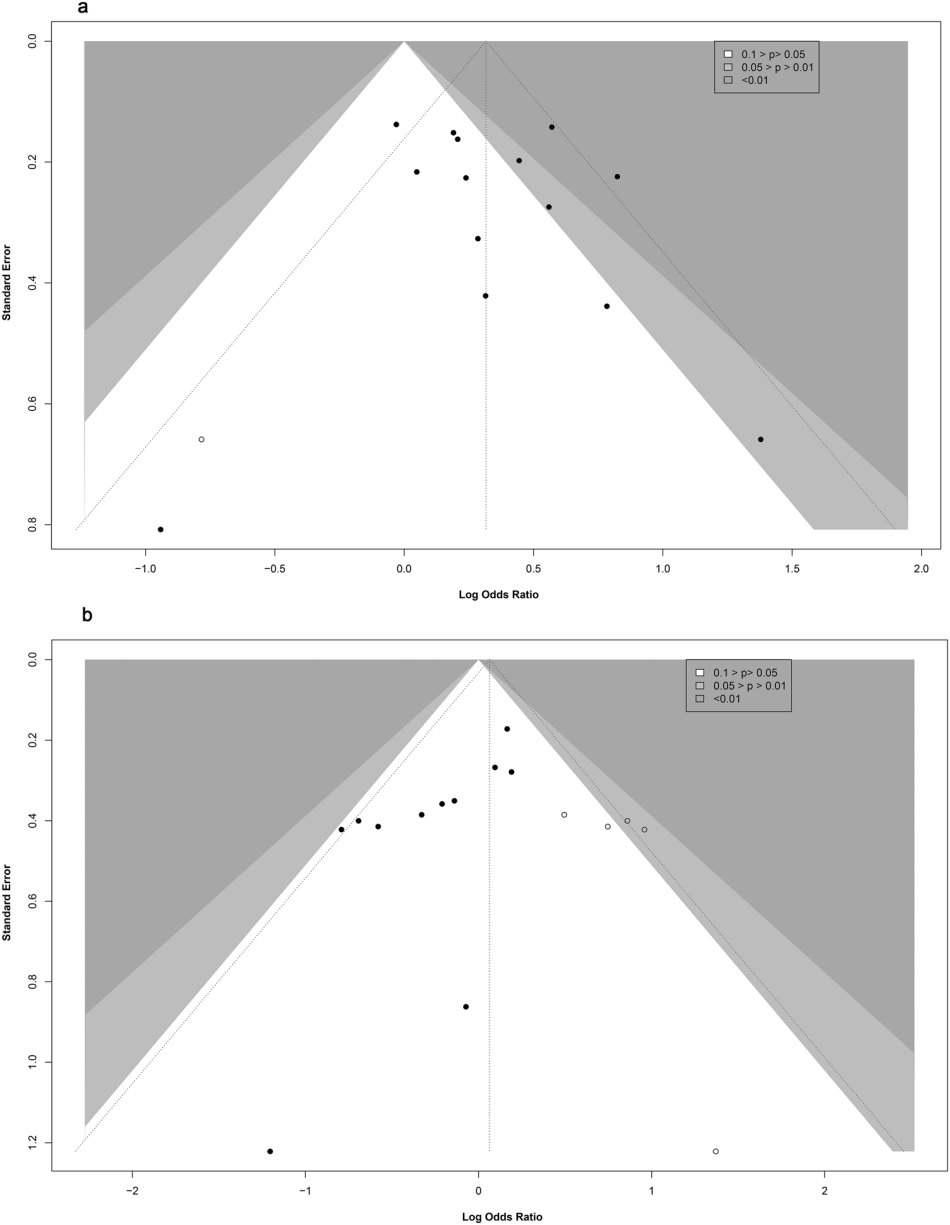
the funnel plot of primary outcomes of crude data about observational studies. (**a**) FI. (**b**) sICH.

As with the above analyses with observational studies, no significant differences were found in the outcomes of observational studies about crude data (Supplemental Fig. 7a, c–f), with the exception of the sICH (Supplemental Fig. 7b). When excluding the study^71^, the effect of direction changed (OR: 1.11, 95% CI: 0.96–1.29). Referring to observational studies of adjusted data, there were no discernible variations in the outcomes (Supplemental Fig. 8a–f).

#### Publication bias

For observational studies of crude data, the inspection of contour-enhanced funnel plots showed evidence of asymmetry of outcomes of FI (Fig. 4a), aICH (Supplemental Fig. 9c), and mortality (Supplemental Fig. 9d). No asymmetry was seen in the outcomes of excellent outcomes (Supplemental Fig. 9a), SR (Supplemental Fig. 9b), and sICH (Fig. 4b). However, there was no evidence in the corresponding Peter's statistical tests for funnel plot asymmetry in terms of the outcomes of FI (*P* = 0.06), excellent outcomes (*P* = 0.56), SR (*P* = 0.83), sICH (*P* = 0.89), aICH (*P* = 0.14), and mortality (*P* = 0.21).

The inspection of contour-enhanced funnel plots for observational studies with adjusted data revealed indications of asymmetries in outcomes of FI and sICH (Fig. 5a–b). There was no asymmetry in the mortality results (Supplemental Fig. 10). Additionally, except for sICH (*P* = 0.01), there was no indication of funnel plot asymmetry in the appropriate Egger's statistical tests for the outcomes of FI (*P* = 0.46) or mortality (*P* = 0.67). We did not run the funnel plot, Peter's, or Egger's statistical tests due to the numerous limitations of including RCTs and other observational studies.

## Discussion

In this systematic review and meta-analysis, a total of approximately 20,000 patients were included in the final analysis. Overall, MT + IVT treatment significantly improved FI, excellent outcomes, and mortality risk in the observational studies, both in raw and adjusted data. Furthermore, it is crucial to note that although in crude analysis we observed an increased risk of sICH and aICH with MT + IVT treatment, no significant difference was found in the adjusted analysis. In the RCTs, we found that MT + IVT treatment reduced the risk of mortality but did not increase the risk of sICH in either the crude or adjusted analyses. Similar effect size directions emerged in the raw and adjusted data in the FI, excellent outcome, and SR domains, implying that there was no significant difference between the two therapies. In addition, although MT + IVT treatment significantly increased the risk of aICH in the raw data, it was not present in the adjusted data. Clearly, the adjusted analysis was more plausible due to the controlled covariates. The use of IVT prior to MT was previously thought to enhance the likelihood of HT^58,72^. However, our results provided further evidence that MT + IVT treatment did not significantly increase the risk of HT. Particularly, adjusted data from observational studies and RCTs, were used to draw conclusions.

The quality of life of impaired patients after stroke was significantly reduced, which caused mental and physical trauma to them and their families as well as a huge economic burden to the public health system. As such, improving the FI of stroke patients was a major rehabilitation object. In this meta-analysis, we found that the MT + IVT group significantly improved the FI in observational studies. although the outcome of FI was at the margin of significance in RCTs. This may be caused by the small number of included RCTs.

Considering the current inconsistency of large RCTs across different study areas, including Asia^23,25,26,28^ and the Europe^9,21,22,24,27^, as well as a study pointing to regionally relevant differences in the safety of IVT treatment in patients with AIS^73^. We performed a subgroup analysis by study area in the meta-analysis. The results indicated that the differences between countries appeared in the outcomes of FI and mortality in RCTs. Additionally, similar results were seen in observational studies (adjusted data) about SR. Overall, European outcomes were better than Asian. Specifically, European studies using MT + IVT therapy showed better FI, higher rates of SR, and lower mortality rates. The findings may suggest that in addition to taking racial factors into account when using MT + IVT therapy, larger clinical research will also be necessary in the future.

Supplemental Table 5 provided a detailed comparison of the prior meta-analysis and the current study. We conducted the most thorough research in this paper, utilizing the largest number of pertinent studies and populations. In addition, crude and adjusted analyses were conducted to further enhance the validity of our findings. Of particular note, although we conducted subgroup analyses by study design and area only, these analyses were based on extracting available data directly from the included studies with the aim of minimizing randomization and sampling error. The primary efficacy results derived from the analysis of observational studies in our study were consistent with previous studies^74^. Regarding the outcomes of the FI and sICH between two regimens by evaluating the raw data, non-significances were both seen when comparing the findings of synthesizing RCTs with the meta-analysis carried out by Vidale and colleagues^14^. Notably, our analysis of the adjusted data revealed that the MT + IVT therapy considerably outperformed the MT alone therapy in terms of excellent outcomes, SR, and mortality.

Several strengths of this study should be noted, and the following were some of the benefits of this study. First, the breadth of the chosen research—observational studies and RCTs with sizable sample sizes—allowed us to perform joint and subgroup analyses and improve statistical analysis. Second, we conducted crude and adjusted data analyses, which increased the credibility of the findings by accounting for confounding factors. Third, except for the outcomes of FI, we also assessed the excellent outcomes (mRS score: 0–1).

However, some limitations must be remarked upon. First, we routinely followed current clinical guidelines so that we only included AIS patients with occlusion of anterior circulation. But there was a need to know whether the MT therapy would be effective for posterior circulation occlusion. However, few studies were seen in this field after searching for literature. Second, because most of the included studies did not provide adjusted data, we performed adjusted analysis by synthesizing only a portion of the included studies, suggesting that the adjusted data were insufficient. Moreover, the number of covariates varied across studies. Third, we only conducted subgroup analyses of study design and area in order to minimize the bias. This may make it challenging for us to investigate additional potential confounders.

## Conclusion

In summary, our findings showed that the MT + IVT therapy did, in fact, raise the rate of SR and lower the risk of mortality. Furthermore, we demonstrated that the MT + IVT therapy did not increase the risk of HT compared with the MT alone therapy. Based on the findings of observational studies, we thought that the MT + IVT therapy was more beneficial in achieving the object of FI. Although the results of FI in RCTs showed the same trend, they formally failed to achieve statistical significance. This would obviously call for further RCTs and analysis, both of which are necessary for future work.

## Supplementary Information

Supplementary Information.

## Data Availability

All data generated or analyzed during this study are included in this published article and its supplementary information files.

## References

[1] GBD 2016 Stroke Collaborators. Global, regional, and national burden of stroke, 1990–2016: A systematic analysis for the Global Burden of Disease Study 2016. *Lancet Neurol.* **18**, 439–458 (2019). 10.1016/S1474-4422(19)30034-1 PMC6494974 30871944

[2] GBD 2019 Stroke Collaborators. Global, regional, and national burden of stroke and its risk factors, 1990–2019: A systematic analysis for the Global Burden of Disease Study 2019. *Lancet Neurol.* **20**, 795–820 (2021). 10.1016/S1474-4422(21)00252-0 PMC8443449 34487721

[3] Powers WJ (2019). Guidelines for the early management of patients with acute ischemic stroke: 2019 update to the 2018 guidelines for the early management of acute ischemic stroke: A guideline for healthcare professionals from the american heart association/American stroke association. Stroke.

[4] Goyal M (2016). Endovascular thrombectomy after large-vessel ischaemic stroke: A meta-analysis of individual patient data from five randomised trials. Lancet.

[5] Goyal N (2018). Comparative safety and efficacy of combined IVT and MT with direct MT in large vessel occlusion. Neurology.

[6] Di Maria F (2018). Intravenous thrombolysis prior to mechanical thrombectomy in acute ischemic stroke: silver bullet or useless bystander?. J. Stroke.

[7] Casetta I (2019). Combined intravenous and endovascular treatment versus primary mechanical thrombectomy. The Italian registry of endovascular treatment in acute stroke. Int. J. Stroke.

[8] Ferrigno M (2018). Intravenous recombinant tissue-type plasminogen activator: Influence on outcome in anterior circulation ischemic stroke treated by mechanical thrombectomy. Stroke.

[9] Gariel F (2018). Mechanical thrombectomy outcomes with or without intravenous thrombolysis. Stroke.

[10] Shi K (2021). tPA mobilizes immune cells that exacerbate hemorrhagic transformation in stroke. Circ. Res..

[11] Nogueira RG (2015). Predictors and clinical relevance of hemorrhagic transformation after endovascular therapy for anterior circulation large vessel occlusion strokes: A multicenter retrospective analysis of 1122 patients. J. Neurointerv. Surg..

[12] Katsanos AH (2019). Intravenous thrombolysis prior to mechanical thrombectomy in large vessel occlusions. Ann. Neurol..

[13] Podlasek A, Dhillon PS, Butt W, Grunwald IQ, England TJ (2021). Direct mechanical thrombectomy without intravenous thrombolysis versus bridging therapy for acute ischemic stroke: A meta-analysis of randomized controlled trials. Int. J. Stroke.

[14] Vidale S, Romoli M, Clemente Agostoni E (2021). Mechanical thrombectomy with or without thrombolysis: A meta-analysis of RCTs. Acta Neurol. Scand..

[15] Moher D (2009). Preferred reporting items for systematic reviews and meta-analyses: The PRISMA statement (Chinese edition). J. Integr. Med..

[16] von Kummer R (2015). The heidelberg bleeding classification: Classification of bleeding events after ischemic stroke and reperfusion therapy. Stroke.

[17] Hacke W (2008). Thrombolysis with alteplase 3 to 4.5 hours after acute ischemic stroke. N. Engl. J. Med..

[18] Wahlgren N (2007). Thrombolysis with alteplase for acute ischaemic stroke in the safe implementation of thrombolysis in stroke-monitoring study (SITS-MOST): An observational study. Lancet.

[19] Goyal M (2014). 2C or not 2C: Defining an improved revascularization grading scale and the need for standardization of angiography outcomes in stroke trials. J. Neurointerv. Surg..

[20] Wells, G. *et al.* The newcastle-ottawa scale (NOS) for assessing the quality of nonrandomized studies in meta- analysis. 12.

[21] Coutinho JM (2017). Combined intravenous thrombolysis and thrombectomy vs thrombectomy alone for acute ischemic stroke: A pooled analysis of the SWIFT and STAR studies. JAMA Neurol..

[22] Chalos V (2019). Endovascular treatment with or without prior intravenous alteplase for acute ischemic stroke. J. Am. Heart Assoc..

[23] Yang P (2020). Endovascular thrombectomy with or without intravenous alteplase in acute stroke. N. Engl. J. Med..

[24] LeCouffe NE (2021). A randomized trial of intravenous alteplase before endovascular treatment for stroke. N. Engl. J. Med..

[25] Zi W (2021). Effect of endovascular treatment alone vs intravenous alteplase plus endovascular treatment on functional independence in patients with acute ischemic stroke: The DEVT randomized clinical trial. JAMA.

[26] Suzuki K (2021). Effect of mechanical thrombectomy without vs with intravenous thrombolysis on functional outcome among patients with acute ischemic stroke: The SKIP randomized clinical trial. JAMA.

[27] Huu An N (2022). Thrombectomy alone versus bridging therapy in acute ischemic stroke: Preliminary results of an experimental trial. Clin. Ter..

[28] Sakai N (2022). Safety, pharmacokinetics and pharmacodynamics of DS-1040, in combination with thrombectomy, in Japanese Patients with acute ischemic stroke. Clin. Drug Investig..

[29] Dávalos A (2012). Retrospective multicenter study of solitaire FR for revascularization in the treatment of acute ischemic stroke. Stroke.

[30] Pfefferkorn T (2012). Preceding intravenous thrombolysis facilitates endovascular mechanical recanalization in large intracranial artery occlusion. Int. J. Stroke.

[31] Kass-Hout T (2014). Is bridging with intravenous thrombolysis of any benefit in endovascular therapy for acute ischemic stroke?. World Neurosurg..

[32] Leker RR (2018). Direct thrombectomy versus bridging for patients with emergent large-vessel occlusions. Interv. Neurol..

[33] Maier IL (2017). Bridging-therapy with intravenous recombinant tissue plasminogen activator improves functional outcome in patients with endovascular treatment in acute stroke. J. Neurol. Sci..

[34] Guedin P (2015). Prior IV thrombolysis facilitates mechanical thrombectomy in acute ischemic stroke. J. Stroke Cerebrovasc. Dis..

[35] Broeg-Morvay A (2016). Direct mechanical intervention versus combined intravenous and mechanical intervention in large artery anterior circulation stroke: A matched-pairs analysis. Stroke.

[36] Behme D (2016). Intravenous thrombolysis facilitates successful recanalization with stent-retriever mechanical thrombectomy in middle cerebral artery occlusions. J. Stroke Cerebrovasc. Dis..

[37] Abilleira S (2017). Outcomes after direct thrombectomy or combined intravenous and endovascular treatment are not different. Stroke.

[38] Bellwald S (2017). Direct mechanical intervention versus bridging therapy in stroke patients eligible for intravenous thrombolysis: A pooled analysis of 2 registries. Stroke.

[39] Weber R (2017). Comparison of outcome and interventional complication rate in patients with acute stroke treated with mechanical thrombectomy with and without bridging thrombolysis. J. Neurointerv. Surg..

[40] de Alonso Leciñana M (2017). Mechanical thrombectomy in patients with medical contraindications for intravenous thrombolysis: A prospective observational study. J. Neurointerv. Surg..

[41] Froehler MT (2017). Interhospital transfer before thrombectomy is associated with delayed treatment and worse outcome in the STRATIS registry (systematic evaluation of patients treated with neurothrombectomy devices for acute ischemic stroke). Circulation.

[42] Rai AT (2018). Intravenous thrombolysis before endovascular therapy for large vessel strokes can lead to significantly higher hospital costs without improving outcomes. J. Neurointerv. Surg..

[43] Wang H (2017). Direct endovascular treatment: An alternative for bridging therapy in anterior circulation large-vessel occlusion stroke. Eur. J. Neurol..

[44] Wee C-K (2017). Outcomes of endovascular thrombectomy with and without thrombolysis for acute large artery ischaemic stroke at a tertiary stroke centre. Cerebrovasc. Dis. Extra..

[45] Merlino G (2017). Short and long-term outcomes after combined intravenous thrombolysis and mechanical thrombectomy versus direct mechanical thrombectomy: A prospective single-center study. J. Thromb. Thrombolysis.

[46] Park H-K (2017). Preceding intravenous thrombolysis in patients receiving endovascular therapy. Cerebrovasc. Dis..

[47] Choi JH (2018). Comparison of outcomes after mechanical thrombectomy alone or combined with intravenous thrombolysis and mechanical thrombectomy for patients with acute ischemic stroke due to large vessel occlusion. World Neurosurg..

[48] Al-Khaled M (2018). Comparing outcome and recanalization results in patients with anterior circulation stroke following endovascular treatment with and without a treatment with rt-PA: A single-center study. Brain Behav..

[49] Heinrichs A (2018). Relevance of standard intravenous thrombolysis in endovascular stroke therapy of a tertiary stroke center. Acta Neurol. Belg..

[50] Sallustio F (2018). Effect of mechanical thrombectomy alone or in combination with intravenous thrombolysis for acute ischemic stroke. J. Neurol..

[51] Bourcier R (2018). Is bridging therapy still required in stroke due to carotid artery terminus occlusions?. J. Neurointerv. Surg..

[52] Goyal N (2019). Impact of pretreatment with intravenous thrombolysis on reperfusion status in acute strokes treated with mechanical thrombectomy. J. Neurointerv. Surg..

[53] Leker RR, Pikis S, Gomori JM, Cohen JE (2015). Is bridging necessary? A pilot study of bridging versus primary stentriever-based endovascular reperfusion in large anterior circulation strokes. J. Stroke Cerebrovasc. Dis..

[54] Guimarães Rocha M (2019). Primary thrombectomy versus combined mechanical thrombectomy and intravenous thrombolysis in large vessel occlusion acute ischemic stroke. J. Stroke Cerebrovasc. Dis..

[55] Balodis A (2019). Endovascular thrombectomy in anterior circulation stroke and clinical value of bridging with intravenous thrombolysis. Acta Radiol..

[56] Gong L (2019). Bridging therapy versus direct mechanical thrombectomy in patients with acute ischemic stroke due to middle cerebral artery occlusion: A clinical- histological analysis of retrieved thrombi. Cell Transplant..

[57] Maingard J (2019). Outcomes of endovascular thrombectomy with and without bridging thrombolysis for acute large vessel occlusion ischaemic stroke. Intern. Med. J..

[58] Hassan AE (2019). Pre-thrombectomy intravenous thrombolytics are associated with increased hospital bills without improved outcomes compared with mechanical thrombectomy alone. J. Neurointerv. Surg..

[59] Reiff T, Barthel O, Ringleb PA, Pfaff J, Mundiyanapurath S (2020). Safety of mechanical thrombectomy with combined intravenous thrombolysis in stroke treatment 4.5 to 9 hours from symptom onset. J. Stroke Cerebrovasc. Dis..

[60] Yi HJ, Sung JH, Lee DH (2020). Bridging Intravenous thrombolysis before mechanical thrombectomy for large artery occlusion may be detrimental with thrombus fragmentation. Curr. Neurovasc. Res..

[61] Hinsenveld WH (2020). Intravenous thrombolysis is not associated with increased time to endovascular treatment. Cerebrovasc. Dis..

[62] Imbarrato G, Bentley J, Gordhan A (2018). Clinical outcomes of endovascular thrombectomy in tissue plasminogen activator versus non-tissue plasminogen activator patients at primary stroke care centers. J. Neurosci. Rural Pract..

[63] Jian Y (2021). Direct versus bridging mechanical thrombectomy in elderly patients with acute large vessel occlusion: A multicenter cohort study. Clin. Interv. Aging.

[64] Tong X (2021). Thrombectomy versus combined thrombolysis and thrombectomy in patients with acute stroke: A matched-control study. Stroke.

[65] Kandregula S (2021). Direct thrombectomy versus bridging thrombolysis with mechanical thrombectomy in middle cerebral artery stroke: A real-world analysis through national inpatient sample data. Neurosurg. Focus.

[66] Zha M (2021). Bridge mechanical thrombectomy may be a better choice for acute large vessel occlusions. J. Thromb. Thrombolysis.

[67] Machado M (2021). Functional outcome after mechanical thrombectomy with or without previous thrombolysis. J. Stroke Cerebrovasc. Dis..

[68] Platko S (2022). Intravenous thrombolysis prior to mechanical thrombectomy does not affect clinical or procedural outcomes in patients with large vessel occlusion acute ischemic stroke. J. Clin. Neurosci..

[69] Dicpinigaitis AJ (2022). Endovascular thrombectomy with and without preceding intravenous thrombolysis for treatment of large vessel anterior circulation stroke: A cross-sectional analysis of 50,000 patients. J. Neurol. Sci..

[70] Minnerup J (2016). Outcome after thrombectomy and intravenous thrombolysis in patients with acute ischemic stroke: A prospective observational study. Stroke.

[71] Banks JL, Marotta CA (2007). Outcomes validity and reliability of the modified rankin scale: Implications for stroke clinical trials: A literature review and synthesis. Stroke.

[72] Tian B (2022). Clinical and imaging indicators of hemorrhagic transformation in acute ischemic stroke after endovascular thrombectomy. Stroke.

[73] Mehta RH (2014). Race/Ethnic differences in the risk of hemorrhagic complications among patients with ischemic stroke receiving thrombolytic therapy. Stroke.

[74] Katsanos AH (2019). Intravenous thrombolysis prior to mechanical thrombectomy in large vessel occlusions. Ann. Neurol..

