# Supplementary material for: Mechanical thrombectomy combined with intravenous thrombolysis for acute ischemic stroke: a systematic review and meta-analyses
Source: Sci Rep. 2023 May 26;13:8597. doi: 10.1038/s41598-023-35532-7 (PMC10220222; doi:10.1038/s41598-023-35532-7)
Supplement: Supplementary file 1 — Supplementary Information. [file 41598_2023_35532_MOESM1_ESM.docx]

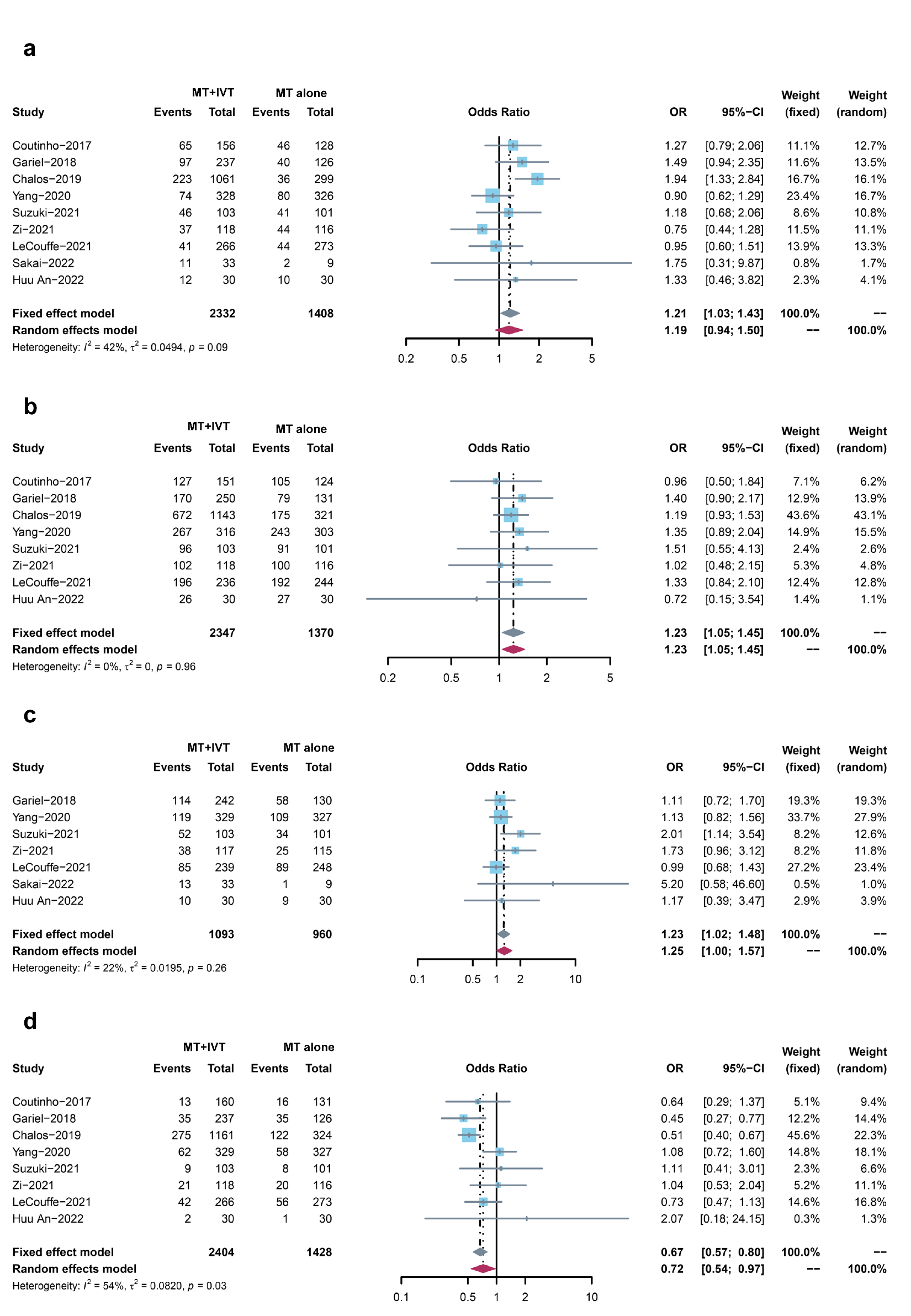


**Supplemental Fig 1** the forest plot of secondary outcomes of crude data about RCTs. **a** excellent outcomes (mRS score: 0-1). **b** SR. **c** aICH. **d** mortality.


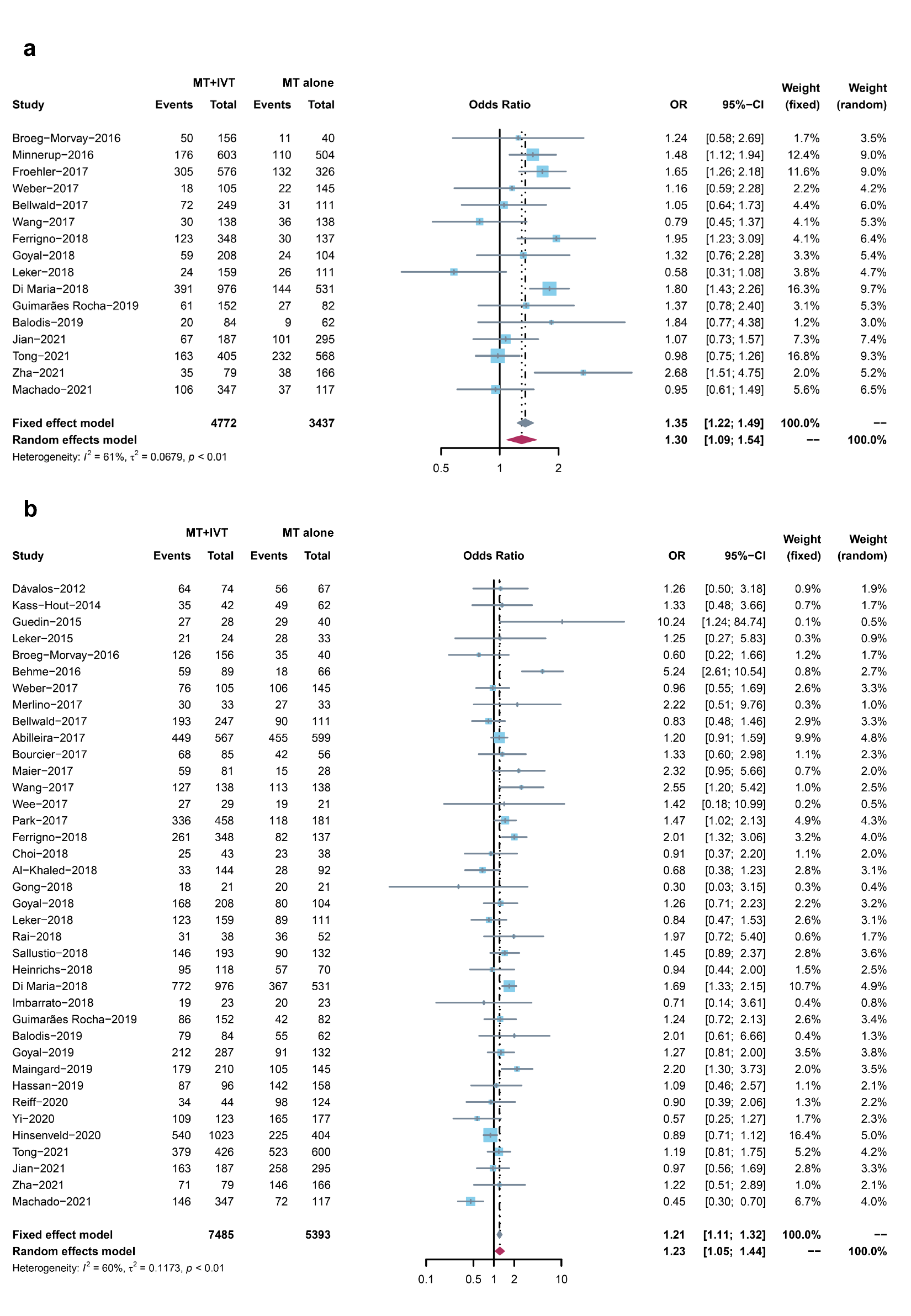


**Supplemental Fig 2** continued


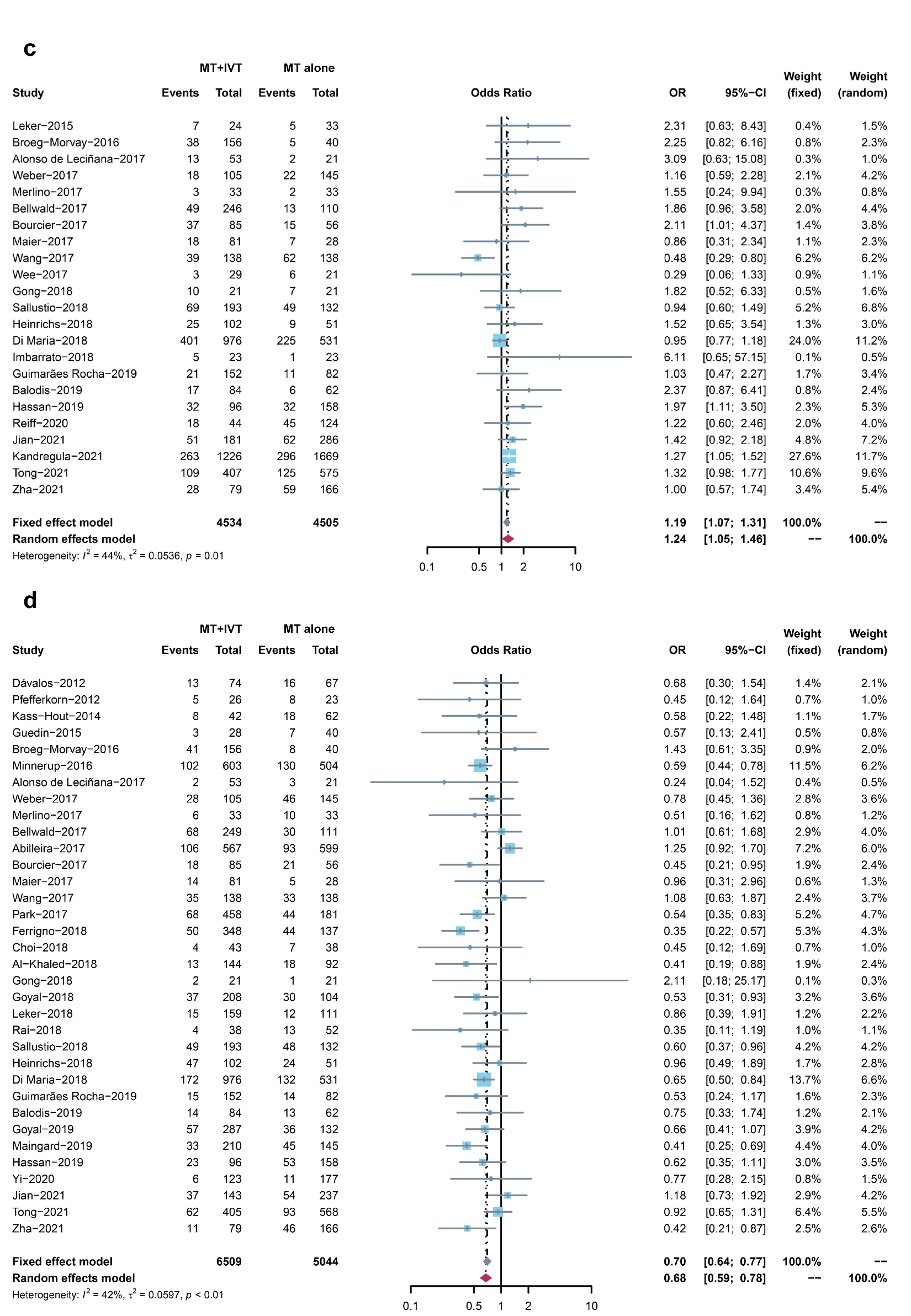


**Supplemental Fig 2** the forest plot of secondary outcomes of crude data about OS. **a** excellent outcomes (mRS score: 0-1). **b** SR. **c** aICH. **d** mortality.


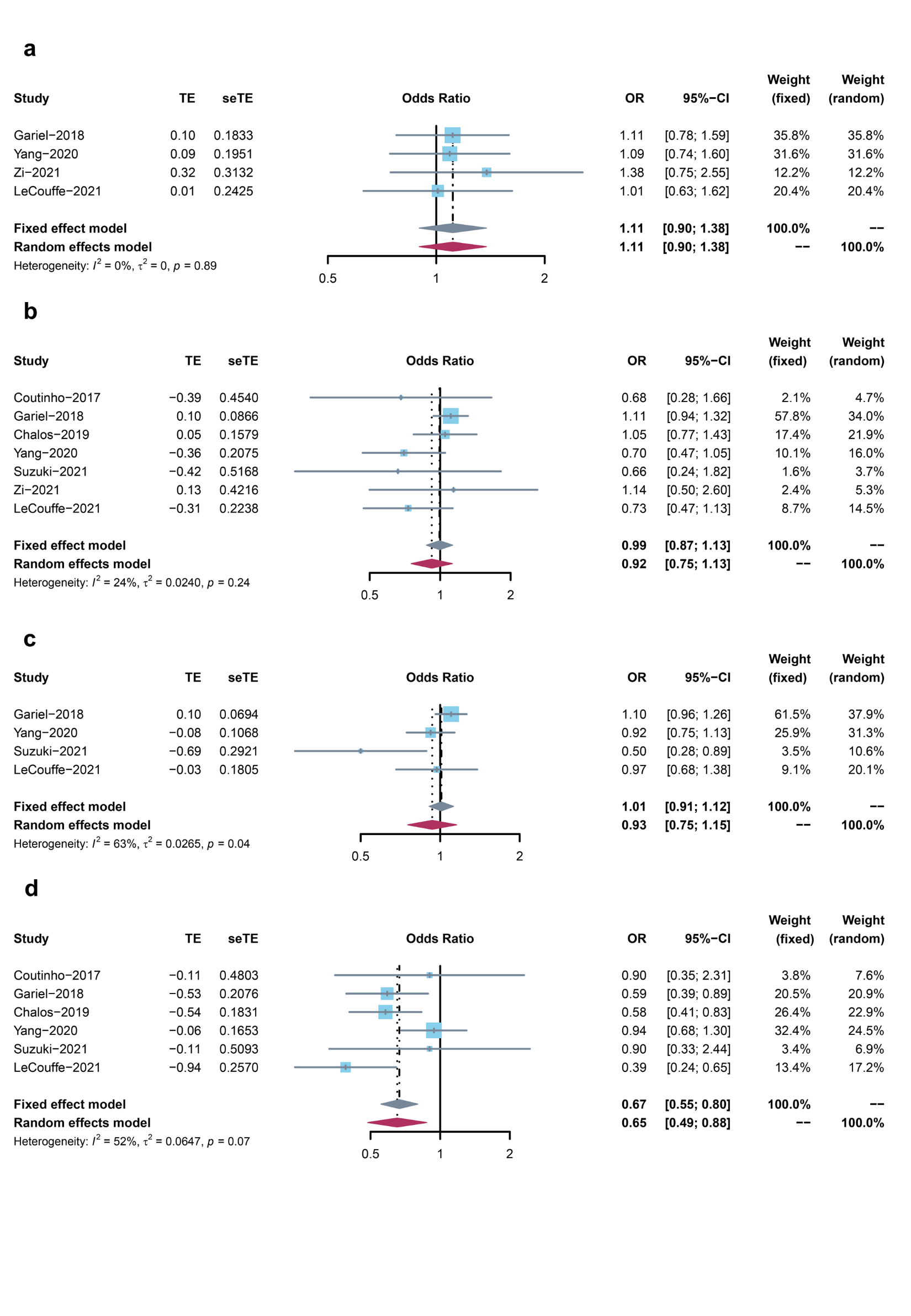


**Supplemental Fig 3** the forest plot of secondary outcomes of adjusted data about RCTs. **a** excellent outcomes (mRS score: 0-1). **b** SR. **c** aICH. **d** mortality.


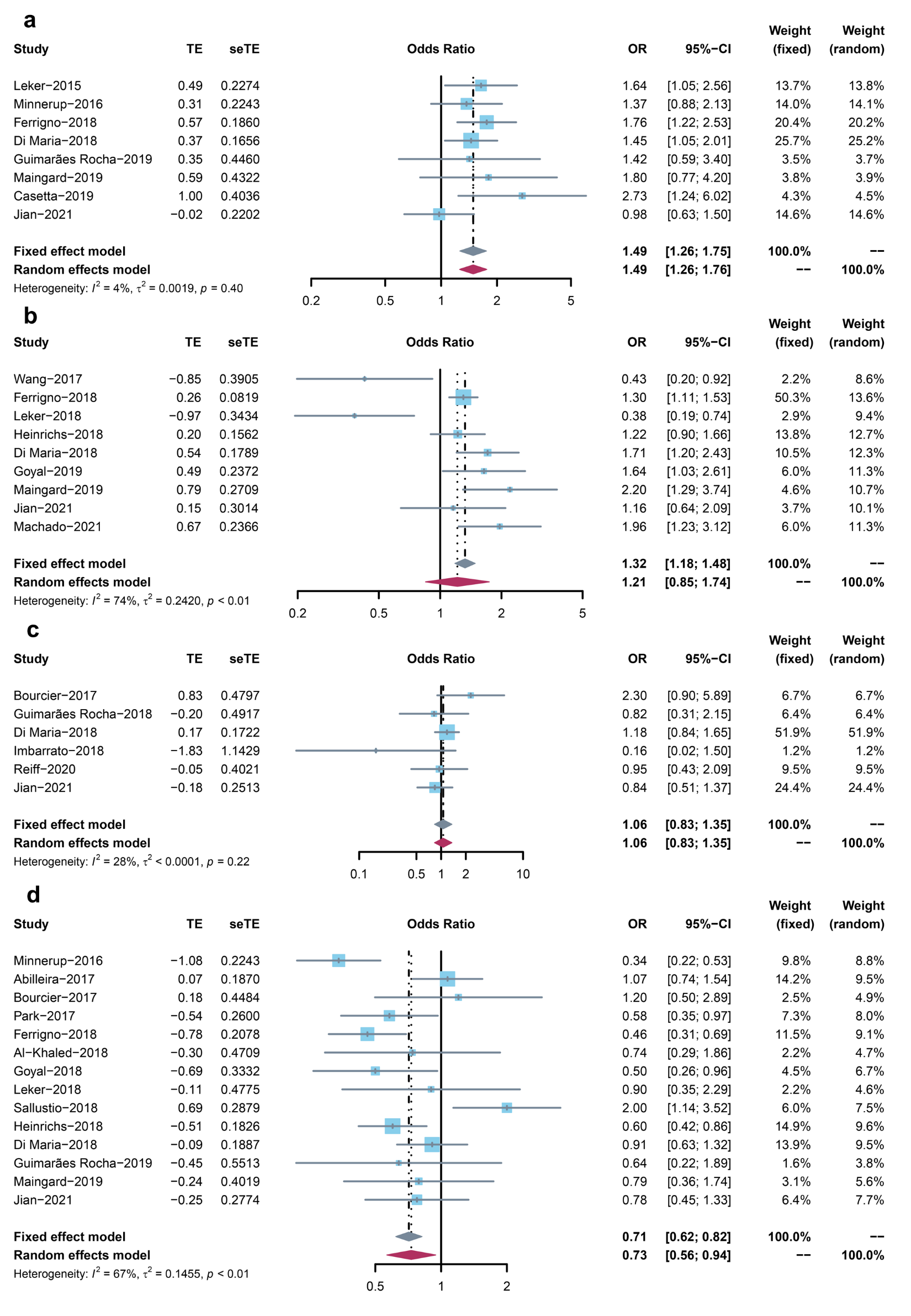


**Supplemental Fig 4** the forest plot of secondary outcomes of adjusted data about OS. **a** excellent outcomes (mRS score: 0-1). **b** SR. **c** aICH. **d** mortality.


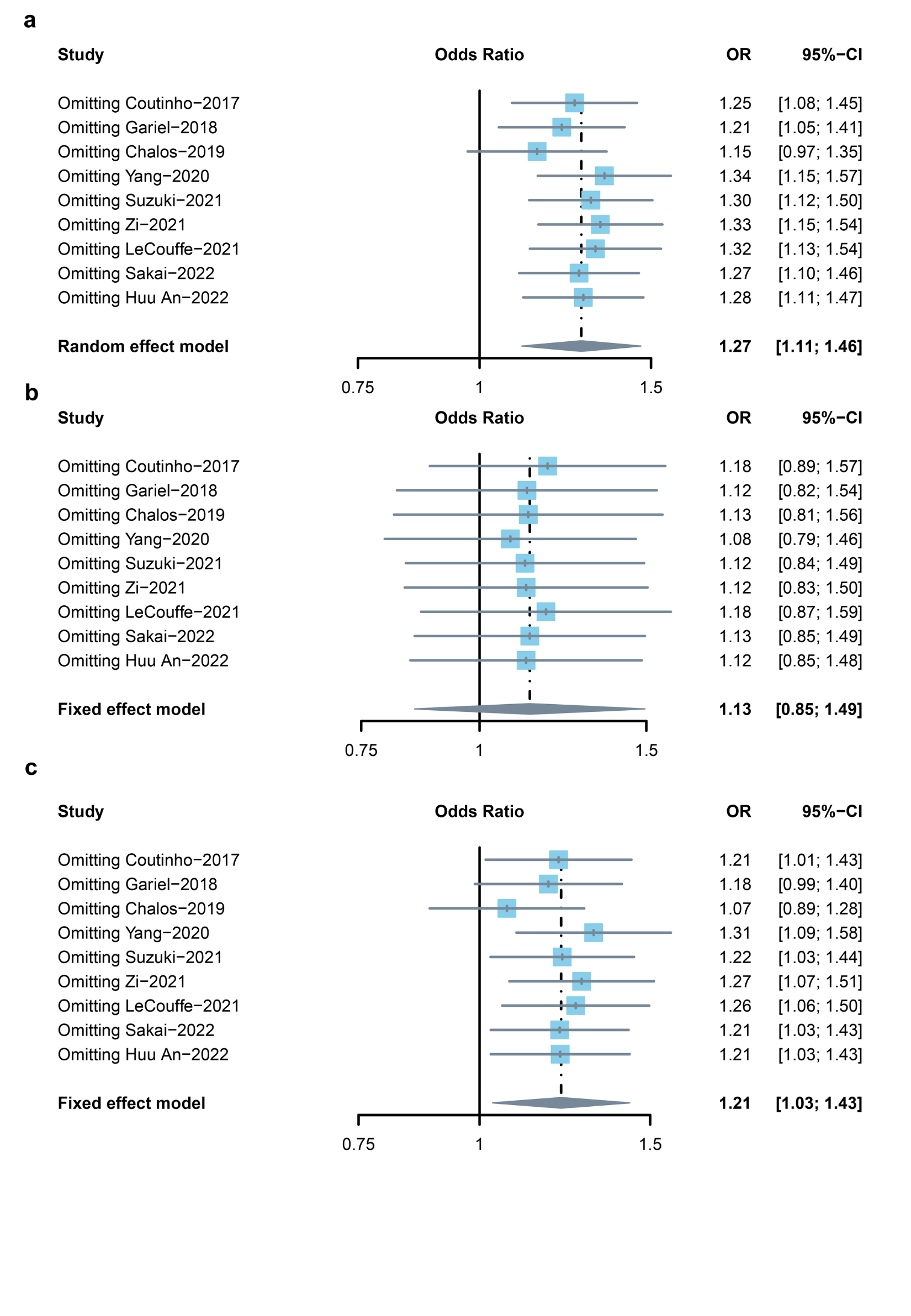


**Supplemental Fig 5** continued


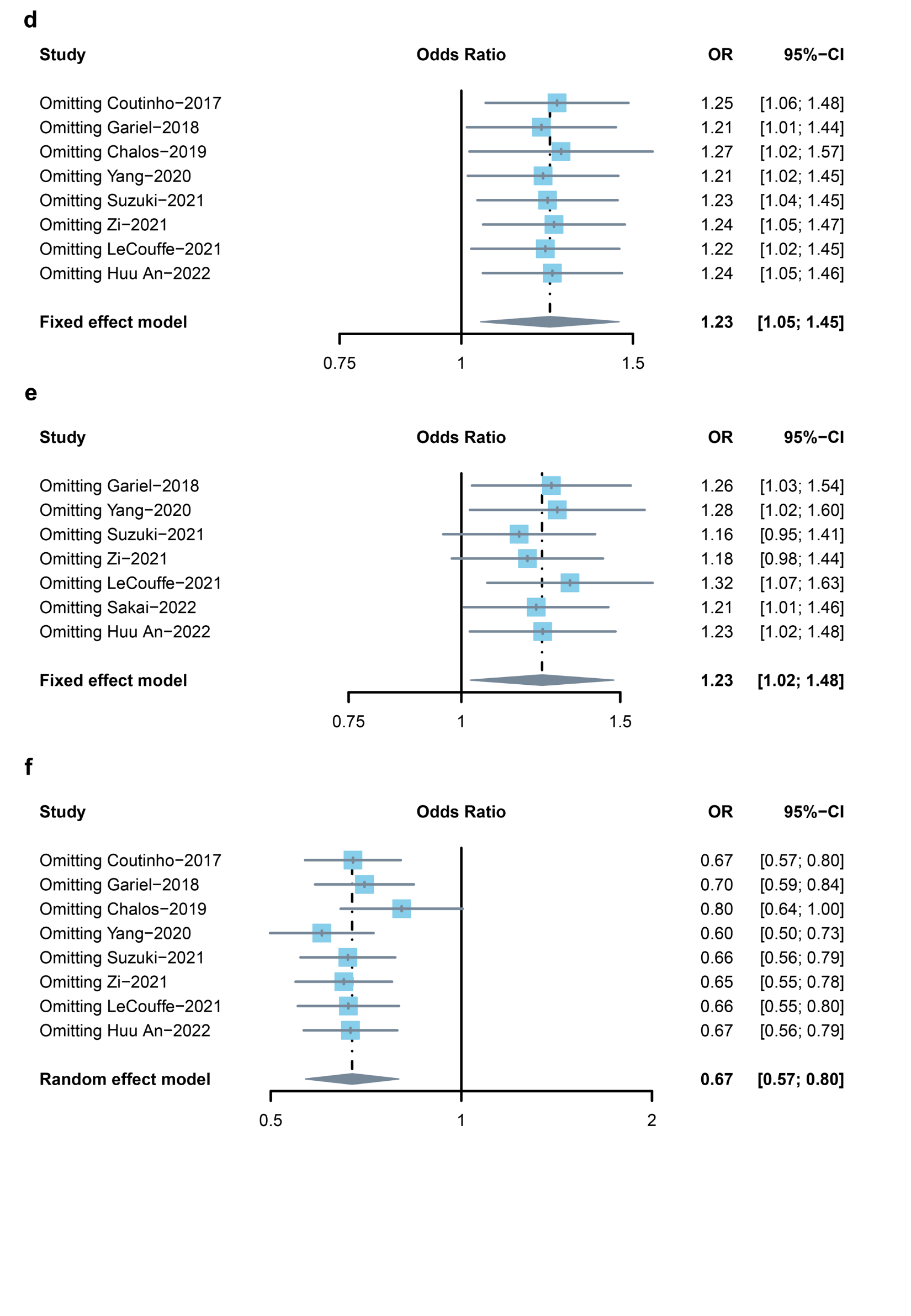


**Supplemental Fig 5** the sensitivity analysis about the outcome of crude data of RCTs. **a** FI. **b** sICH. **c** excellent outcomes (mRS score: 0-1). **d** SR. **e** aICH. **f** mortality.


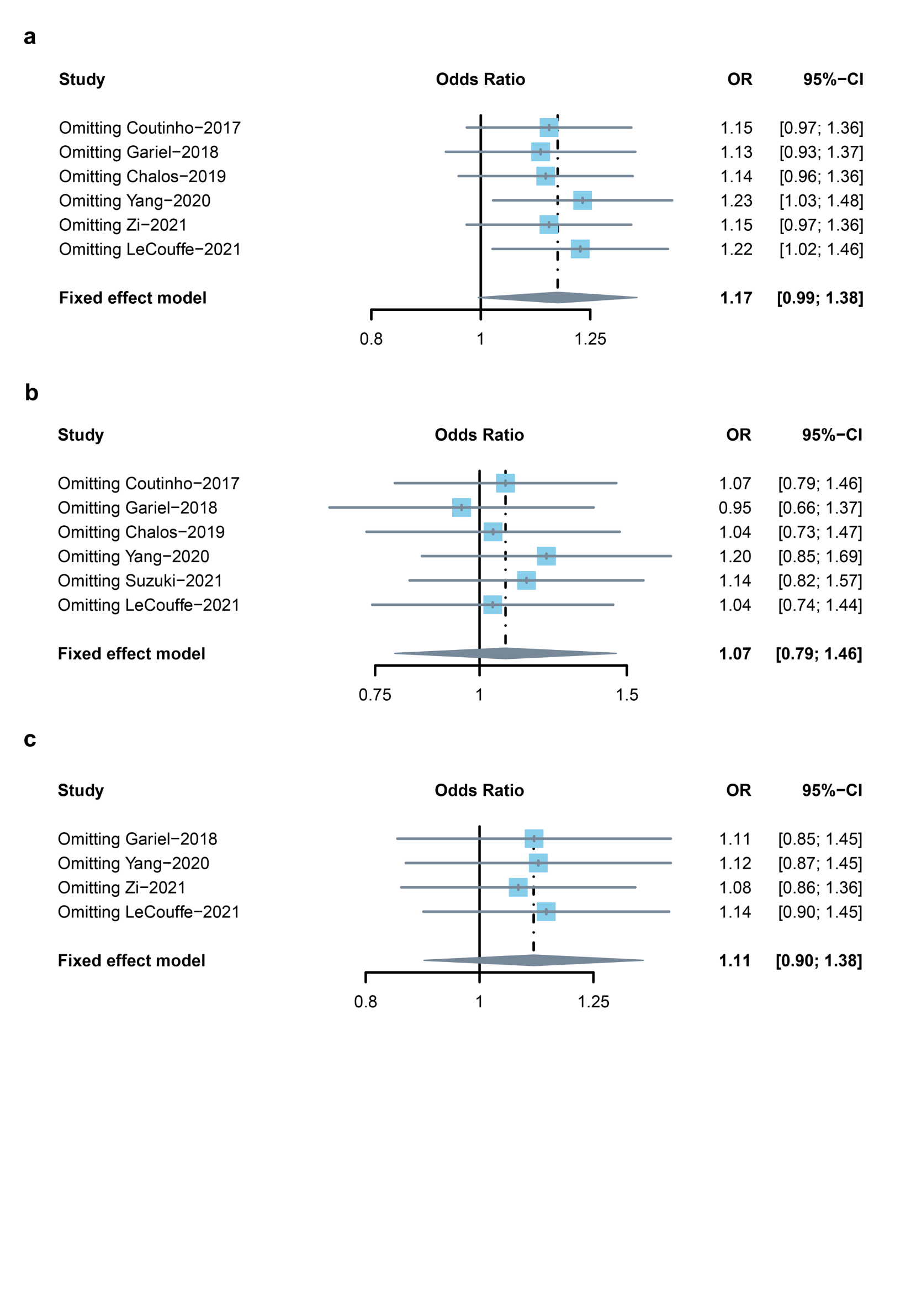


**Supplemental Fig 6** continued


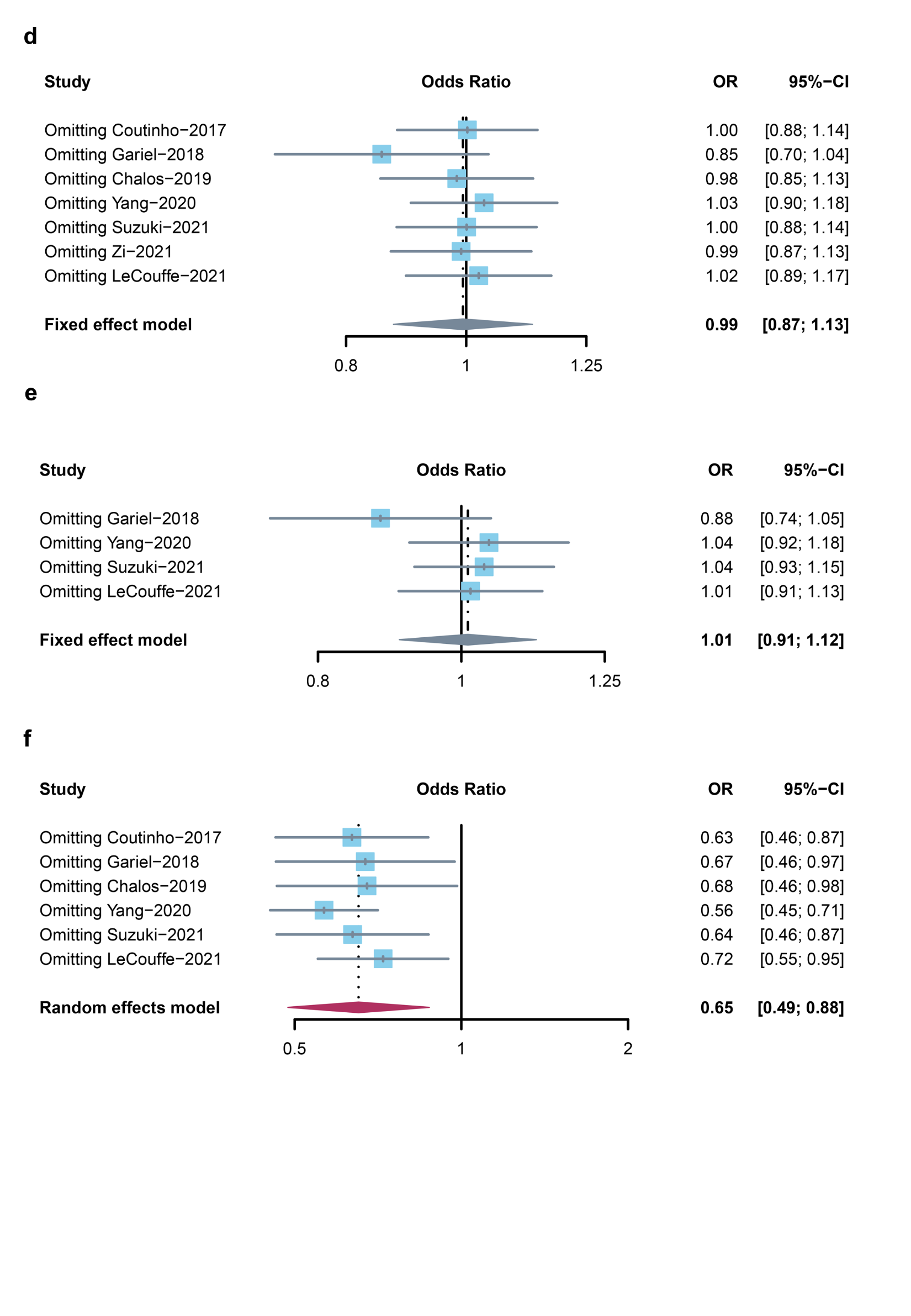


**Supplemental Fig 6** the sensitivity analysis about the outcome of adjusted data of RCTs. **a** FI. **b** sICH. **c** excellent outcomes (mRS score: 0-1). **d** SR. **e** aICH. **f** mortality.


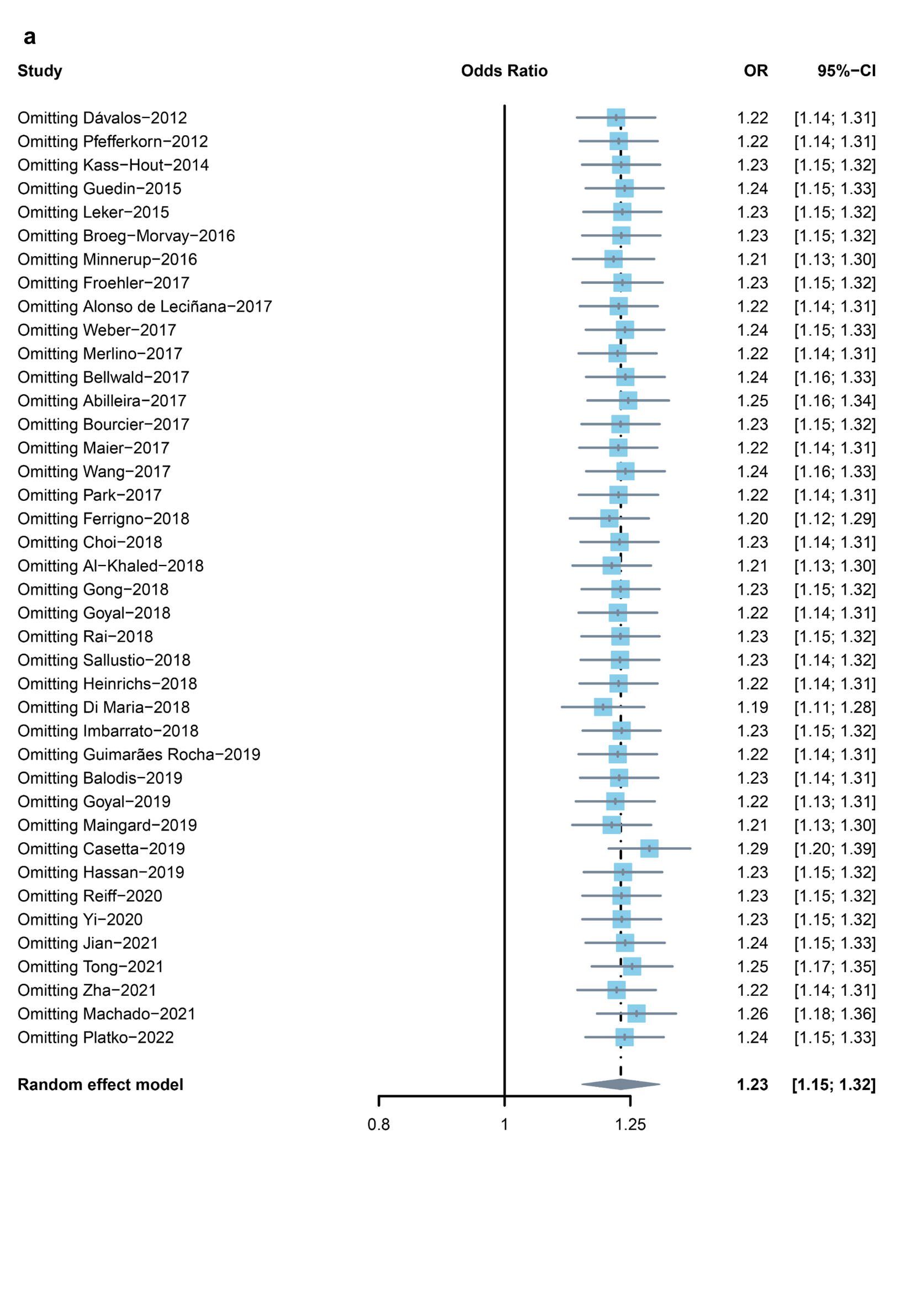


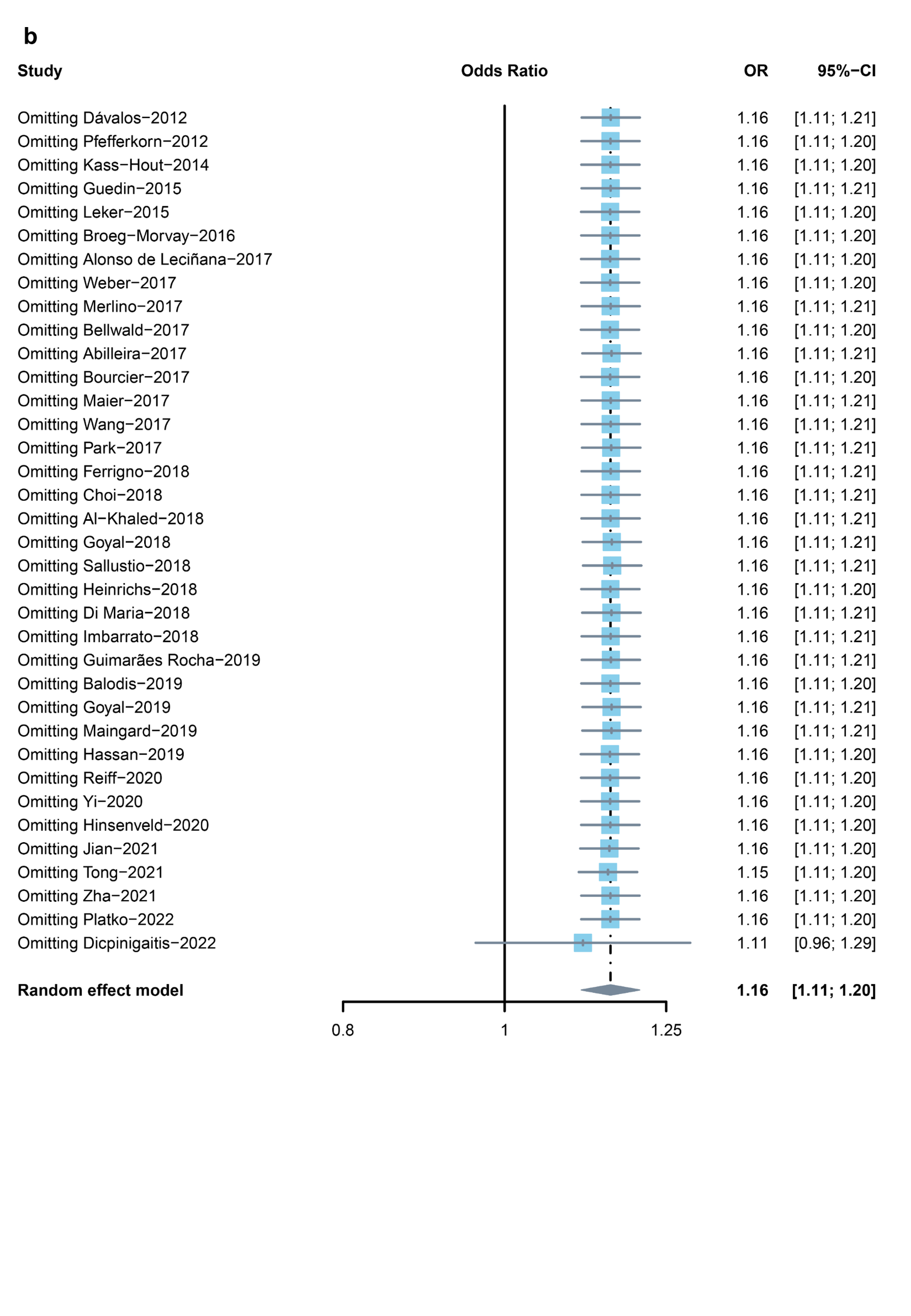


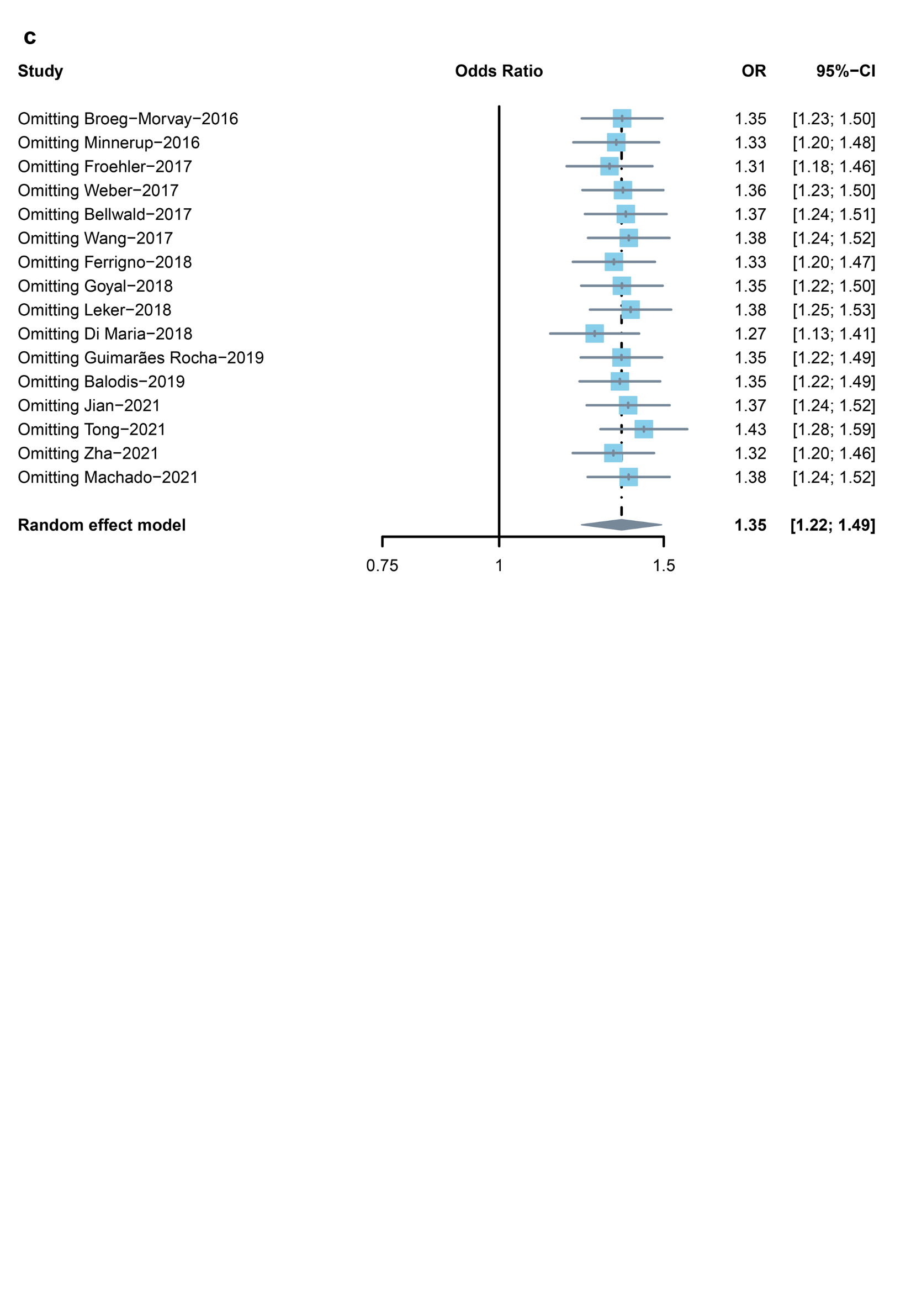


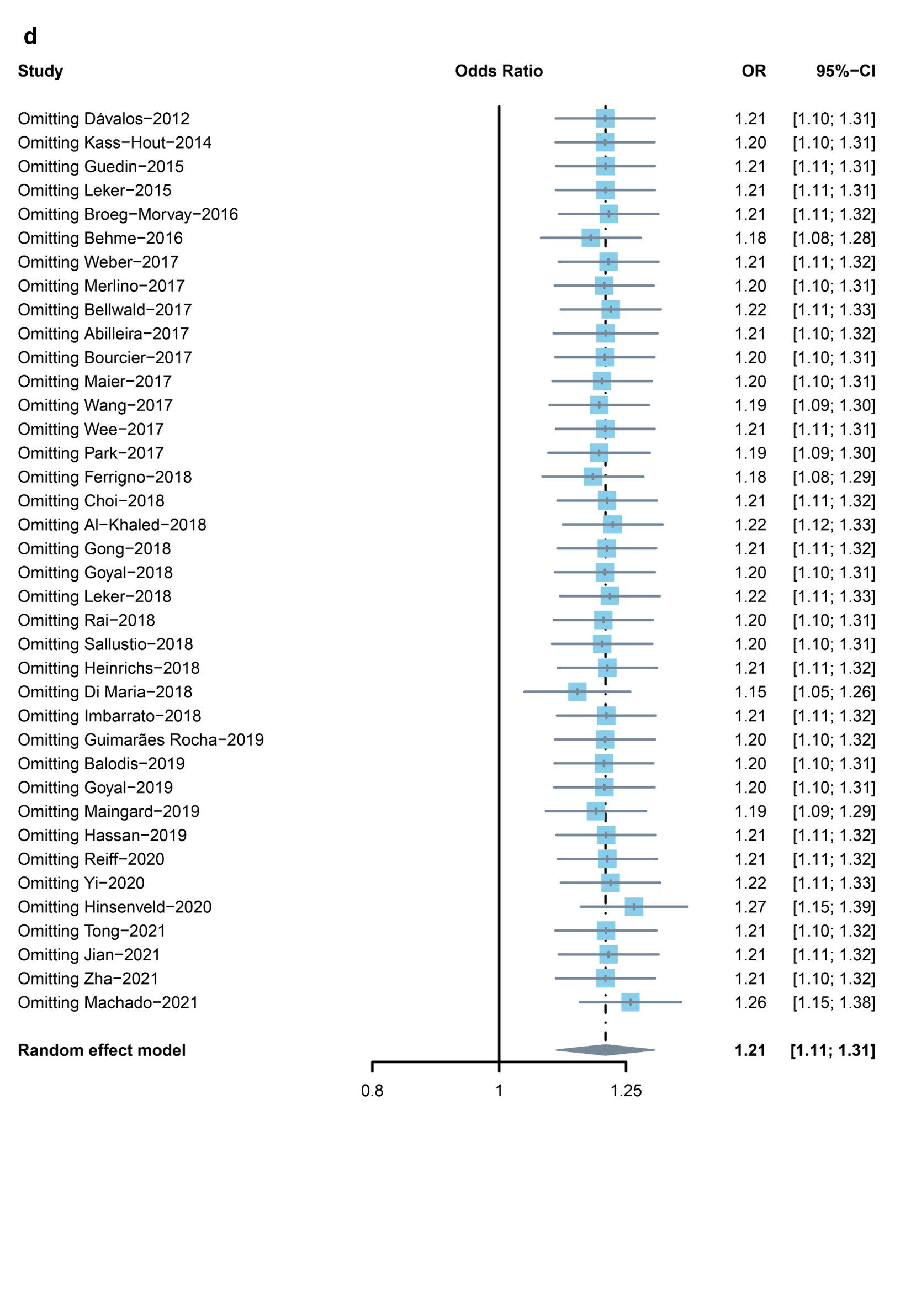


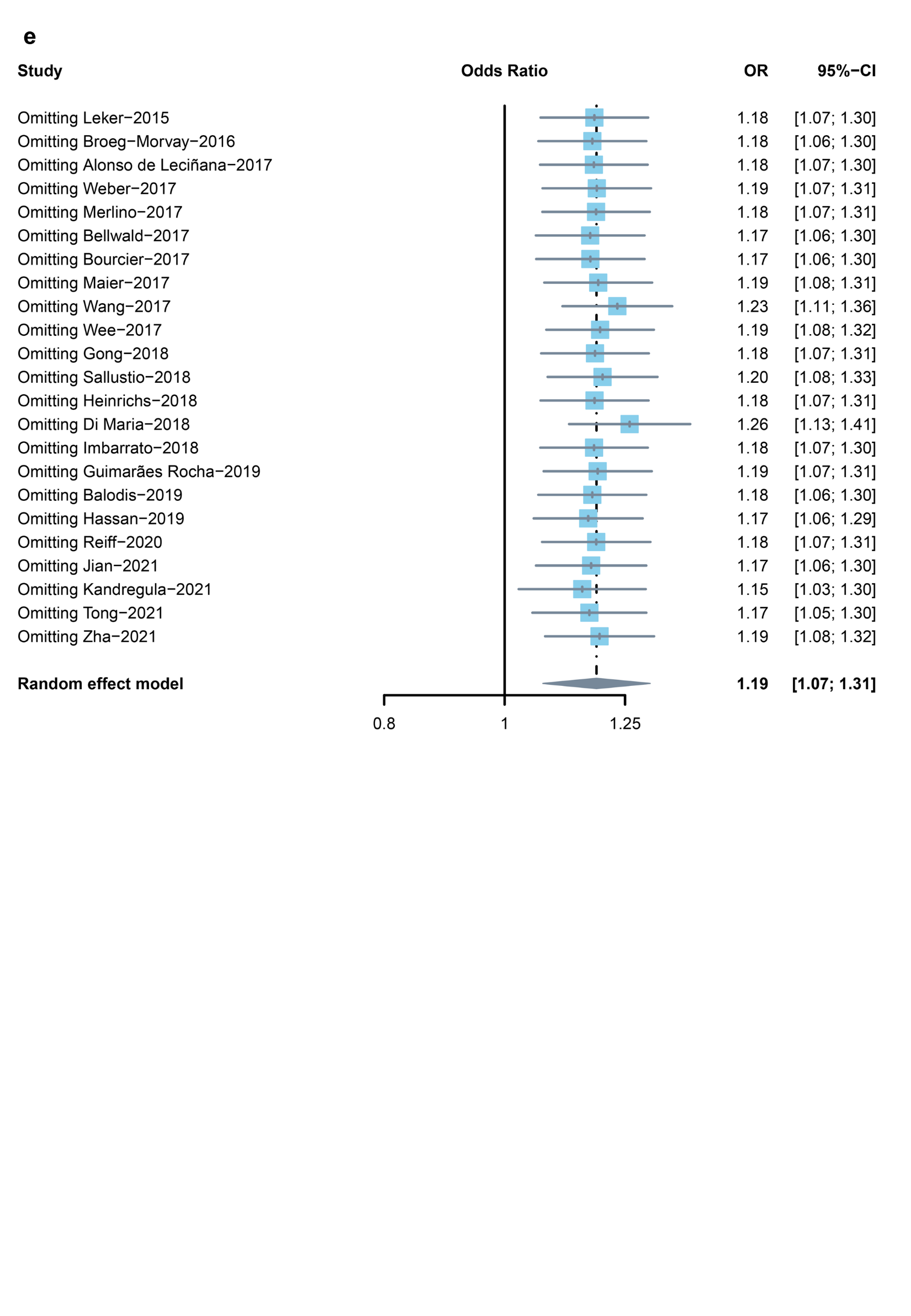


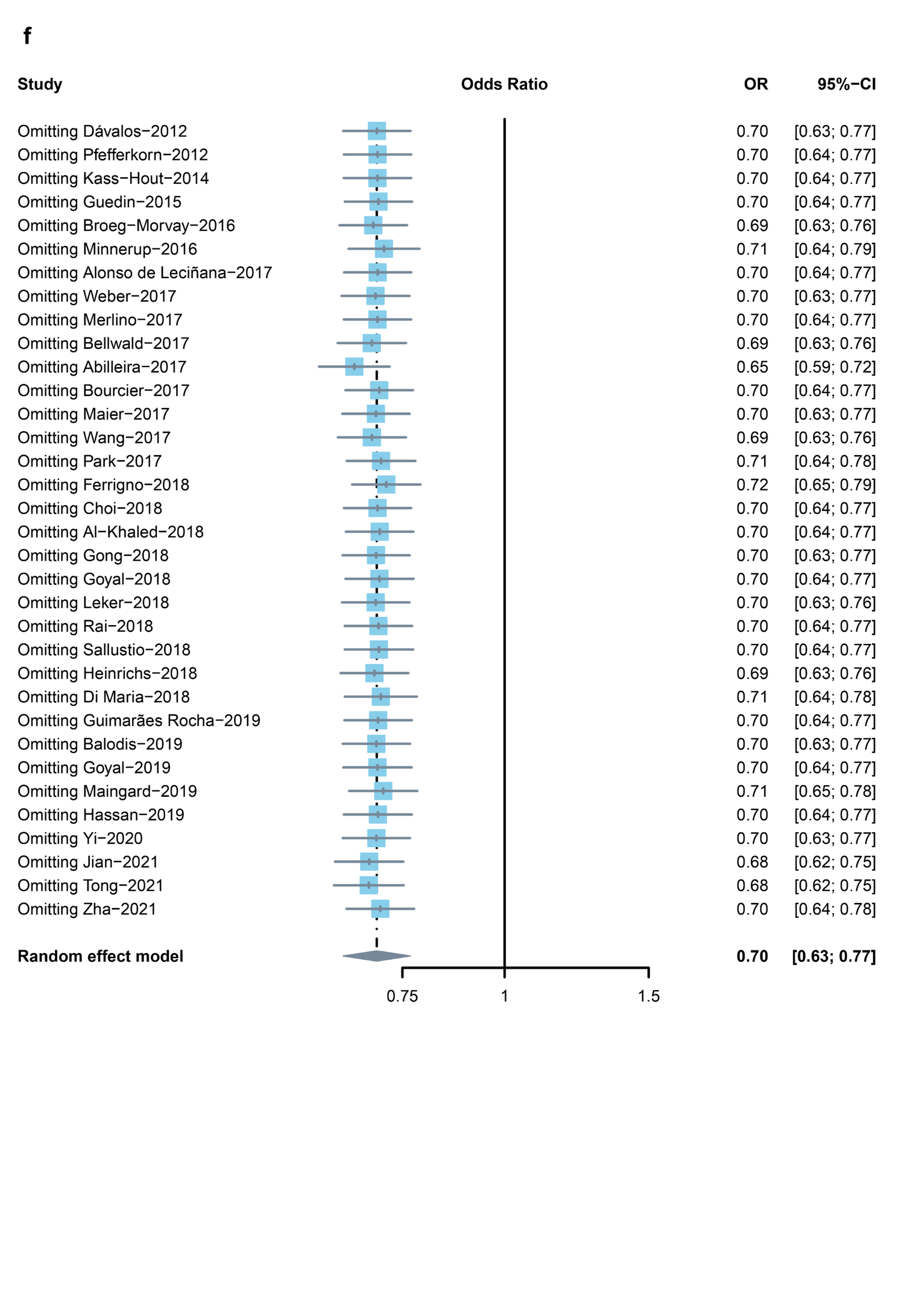


**Supplemental Fig 7** the sensitivity analysis about the outcome of crude data of OS. **a** FI. **b** sICH. **c** excellent outcomes (mRS score: 0-1). **d** SR. **e** aICH. **f** mortality.


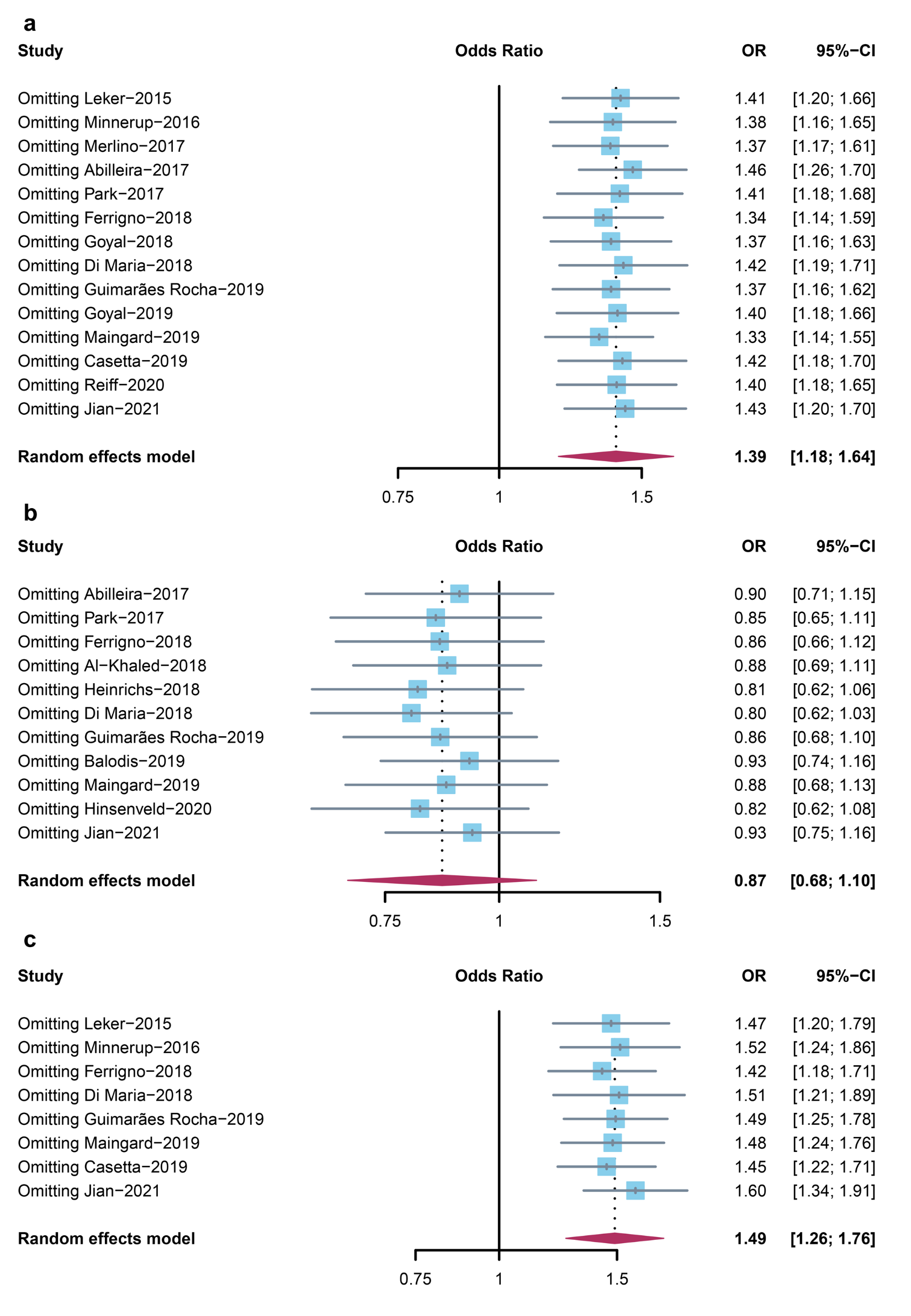


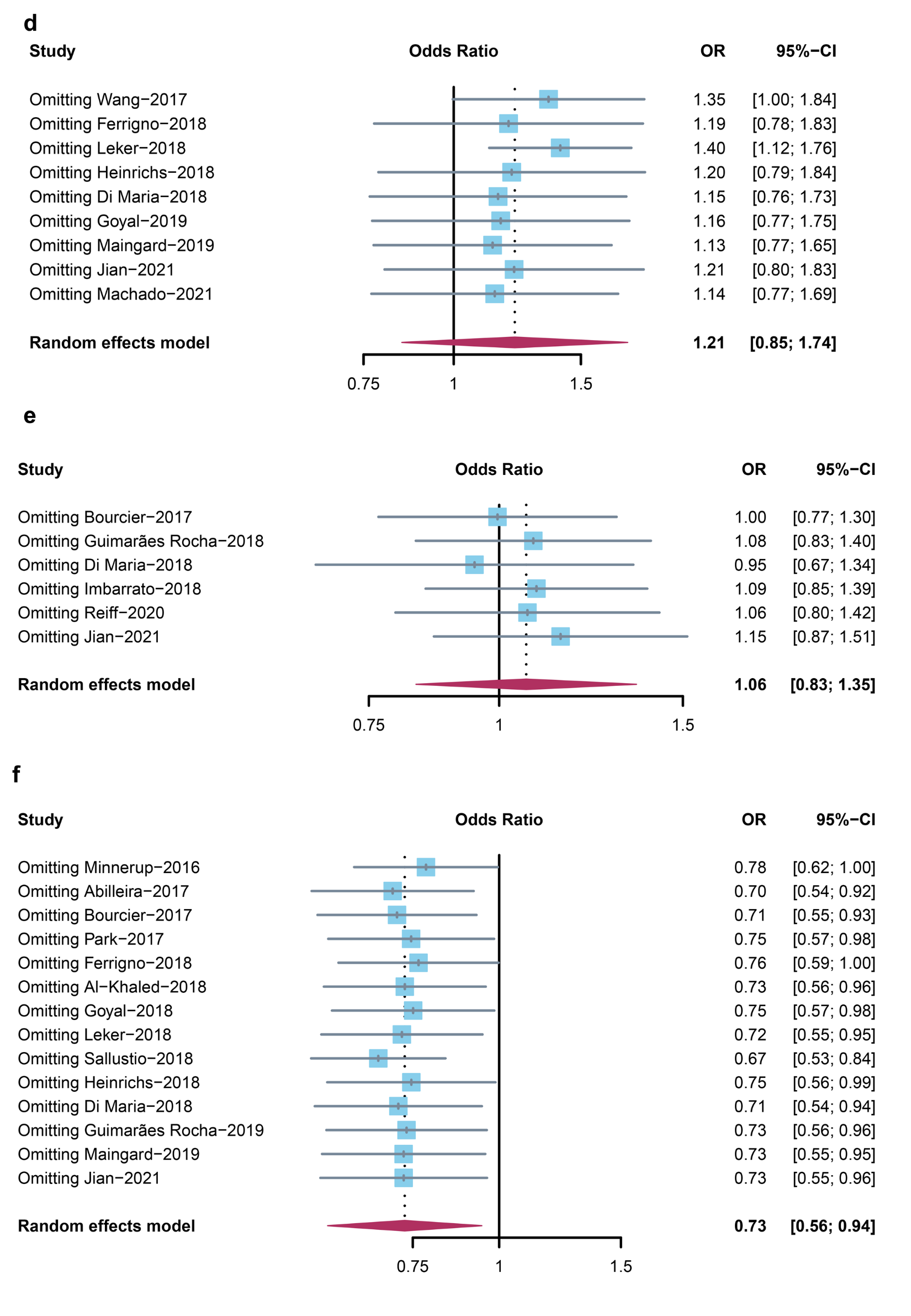


**Supplemental Fig 8** the sensitivity analysis about the outcome of adjusted data of OS. **a** FI. **b** sICH. **c** excellent outcomes (mRS score: 0-1). **d** SR. **e** aICH. **f** mortality.


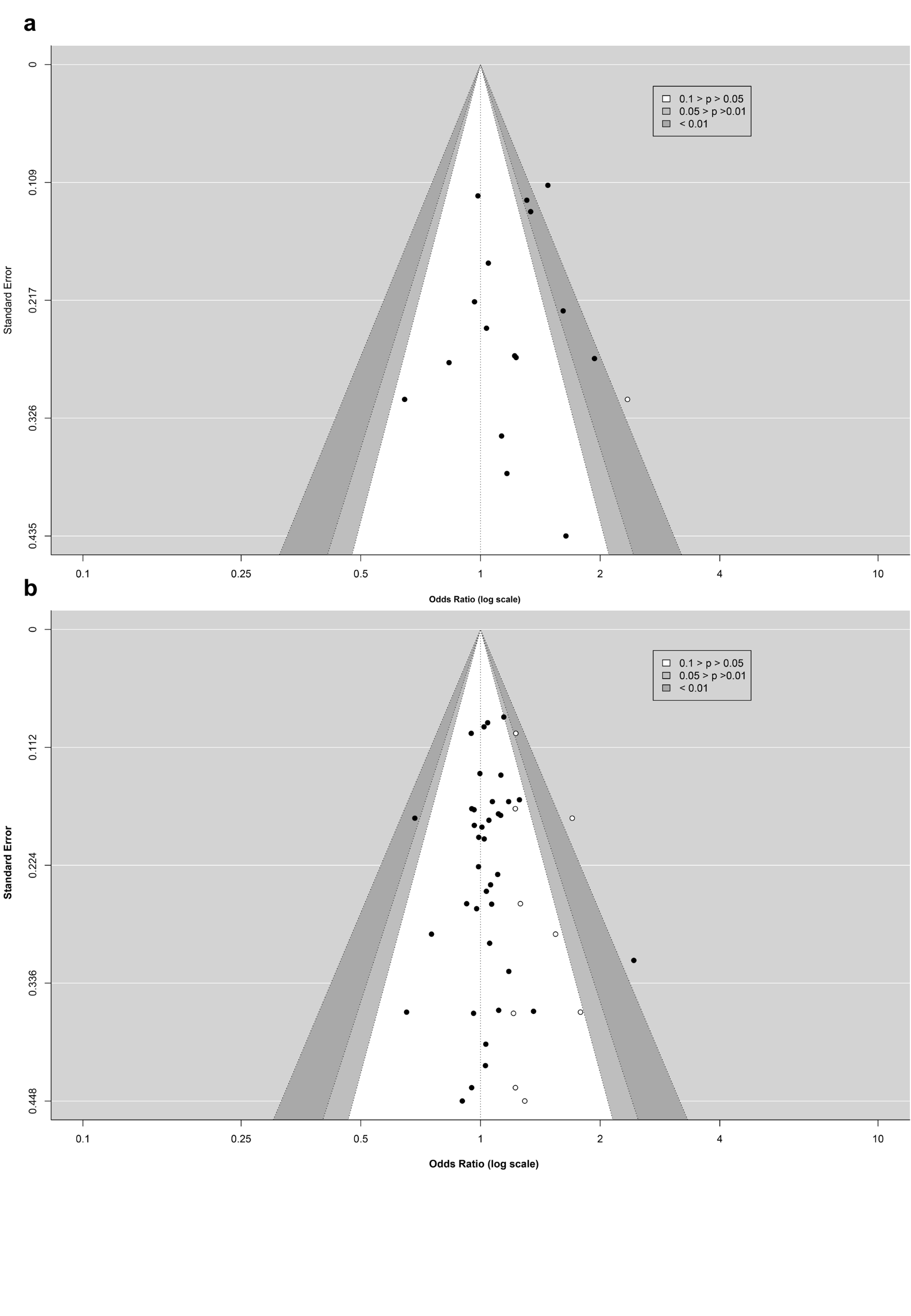


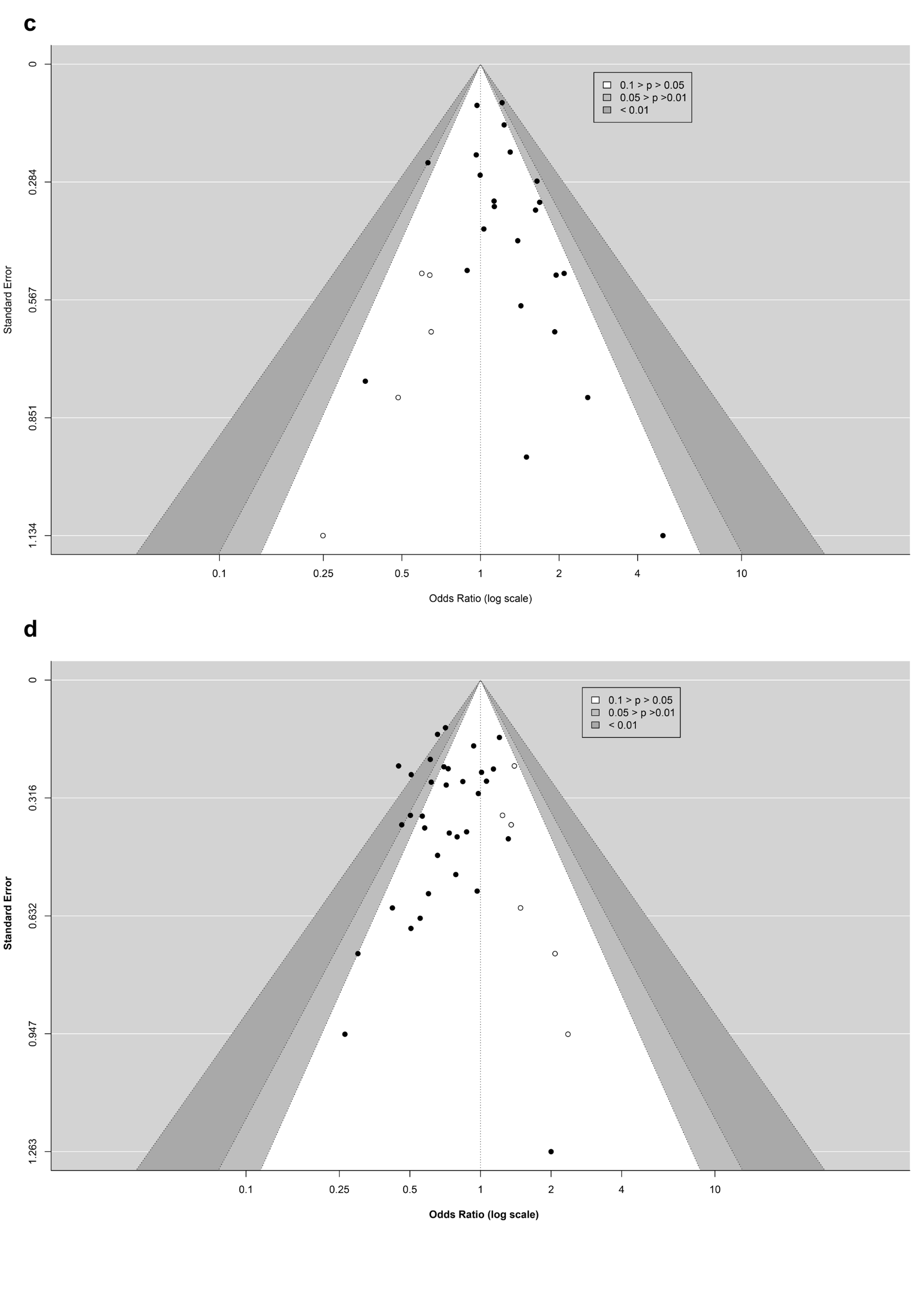


**Supplemental Fig 9** the funnel plot about the secondary outcome of crude data of OS. **a** excellent outcomes (mRS score: 0-1). **b** SR. **c** aICH. **d** mortality.


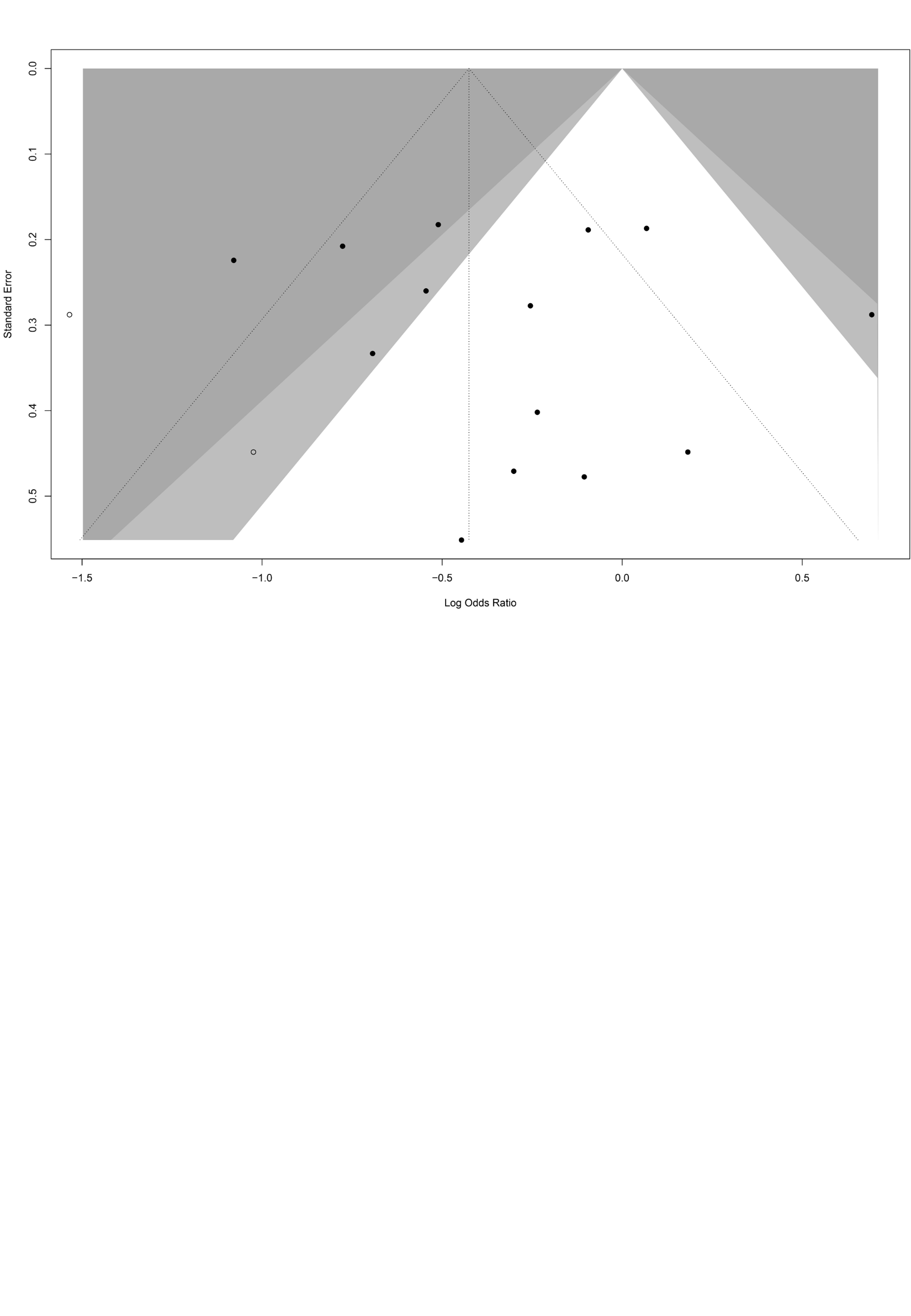


**Supplemental Fig 10** the funnel plot about the outcome of mortality of adjusted data OS.


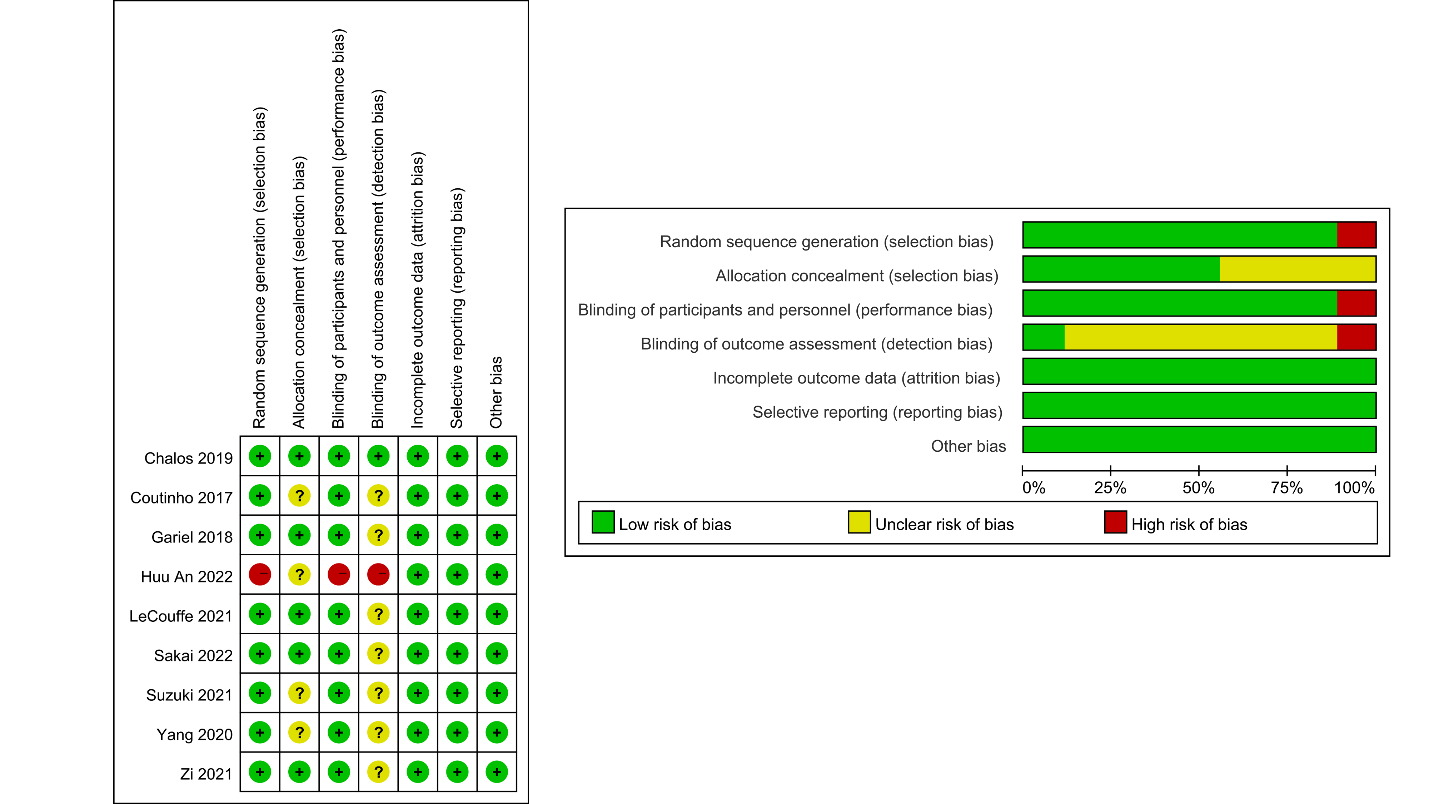


**Supplemental Fig 11** assessment of quality using the RoB (RCTs)

**Supplemental Table1** Subgroup analysis in RCTs

| Outcomes | Crude data | | |
| --- | --- | --- | --- |
| Area | North America | Europe | Asia |
| FI | | | |
| No of studies | 1 | 4 | 4 |
| OR and 95% CI | 1.50 (0.94-2.40) | 1.46 (1.07-1.98) | 0.95 (0.75-1.21) |
| Heterogeneity | - | 505 | 0 |
| χ^2^ | 5.99 | | |
| *P*-value | **0.05** | | |
| sICH | | | |
| No of studies | 1 | 4 | 4 |
| OR and 95% CI | 0.32 (0.06-1.67) | 1.10 (0.70-1.56) | 1.35 (0.83-2.21) |
| Heterogeneity | - | 0 | 0 |
| χ^2^ | 2.78 | | |
| *P*-value | 0.25 | | |
| excellent outcomes (mRS score:0-1) | | | |
| No of studies | 1 | 4 | 4 |
| OR and 95% CI | 1.27(0.79-2.06) | 1.42 (0.99-2.03) | 0.93 (0.71-1.20) |
| Heterogeneity |  |  |  |
| χ^2^ | 3.91 | | |
| *P*-value | 0.14 | | |
| SR | | | |
| No of studies | 1 | 4 | 4 |
| OR and 95% CI | 0.96 (0.50-1.84) | 1.24 (1.02-1.51) | 1.29 (0.91-1.81) |
| Heterogeneity | - | 0 | 0 |
| χ^2^ | 0.64 | | |
| *P*-value | 0.73 | | |
| aICH | | | |
| No of studies | - | 3 | 4 |
| OR and 95% CI | - | 1.04 (0.80-1.37) | 1.54 (1.05-2.27) |
| Heterogeneity |  | 0 | 41% |
| χ^2^ | - | 2.64 | |
| *P*-value | - | 0.10 | |
| Mortality | | | |
| No of studies | 1 | 4 | 3 |
| OR and 95% CI | 0.64 (0.29-1.37) | 0.55 (0.44-0.69) | 1.07 (0.78-1.48) |
| Heterogeneity | - | 13% | 0 |
| χ^2^ | 11.18 | | |
| *P*-value | **＜0.01** | | |

**Supplemental Table 2** Subgroup analysis of study area in observational studies

| Outcomes | Crude data | | | adjusted data | | |
| --- | --- | --- | --- | --- | --- | --- |
| Area | North America | Europe | Asia | North America | Europe | Asia |
| FI | | | | | | |
| No of studies | 8 | 23 | 9 | 2 | 9 | 3 |
| OR and 95% CI | 1.23 (1.00-1.51) | 1.46 (1.13-1.87) | 1.13 (0.93-1.36) | 1.56 (1.03-2.36) | 1.48 (1.19-1.84) | 1.11 (0.82-1.49) |
| Heterogeneity |  |  |  | 0 | 60% | 5% |
| χ^2^ | 2.52 | | | 2.80 | | |
| *P*-value | 0.28 | | | 0.25 | | |
| sICH | | | | | | |
| No of studies | 7 | 22 | 7 | - | 9 | 2 |
| OR and 95% CI | 1.05 (0.67-1.64) | 1.06 (0.87-1.30) | 1.16 (1.12-1.21) | - | 0.93 (0.73-1.18) | 0.65 (0.35-1.23) |
| Heterogeneity |  |  |  | - | 5% | 29% |
| χ^2^ | 0.92 | | | 1.01 | | |
| *P*-value | 0.63 | | | 0.31 | | |
| excellent outcomes (mRS score:0-1) | | | | | | |
| No of studies | 2 | 9 | 5 | - | 6 | 2 |
| OR and 95% CI | 1.58 (1.24-2.02) | 1.43 (1.19-1.73) | 1.05 (0.67-1.66) | - | 1.59 (1.31-1.93) | 1.26 (0.76-2.10) |
| Heterogeneity | 0 | 25% | 74% | - | 0 | 63% |
| χ^2^ | 2.37 | | | - | 0.70 | |
| *P*-value | 0.31 | | | - | 0.40 | |
| SR | | | | | | |
| No of studies | 6 | 21 | 10 | 1 | 5 | 3 |
| OR and 95% CI | 1.25 (0.95-1.71) | 1.25 (0.98-1.61) | 1.40 (0.90-1.45) | 1.21 (0.85-1.74) | 1.51 (0.23-1.86) | 0.59 (0.29-1.21) |
| Heterogeneity | 0 | 75% | 24% | - | 48% | 72% |
| χ^2^ | 0.44 | | | 6.49 | | |
| *P*-value | 0.93 | | | **0.04** | | |
| aICH | | | | | | |
| No of studies | 3 | 13 | 6 | - | - | - |
| OR and 95% CI | 1.55(0.99-2.43) | 1.27 (1.01-1.59) | 1.11 (0.73-1.70) | - | - | - |
| Heterogeneity | 48% | 18% | 67% | - | - | - |
| χ^2^ | 4.76 | | | - | | |
| *P*-value | 0.19 | | | - | | |
| Mortality | | | | | | |
| No of studies | 5 | 21 | 8 | 1 | 10 | 3 |
| OR and 95% CI | 0.59 (0.44-0.78) | 0.65 (0.54-0.79) | 0.79 (0.58-1.07) | 0.73 (0.56-0.94) | 0.76 (0.54-1.08) | 0.69 (0.49-0.98) |
| Heterogeneity | 0 | 53% | 41% | - | 76% | 0 |
| χ^2^ | 1.95 | | | 1.28 | | |
| *P*-value | 0.38 | | | 0.53 | | |

**Supplemental Table 3** Subgroup analysis of study design in observational studies

| Outcomes | Crude data | | adjusted data | |
| --- | --- | --- | --- | --- |
| Study design | Retrospective study | Prospective study | Retrospective study | Prospective study |
| FI | | | | |
| No of studies | 27 | 13 | 8 | 6 |
| OR and 95% CI | 1.20 (0.98-1.47) | 1.51 (1.21-1.88) | 1.39 (1.06-0.83) | 1.41 (1.16-1.72) |
| Heterogeneity | 71% | 65% | 56% | 40% |
| χ^2^ | 2.27 | | 0.01 | |
| *P*-value | 0.13 | | 0.93 | |
| sICH | | | | |
| No of studies | 24 | 12 | 5 | 6 |
| OR and 95% CI | 1.07 (0.92-1.24) | 1.28 (1.01-1.63) | 0.82 (0.56-1.21) | 0.87 (0.63-1.20) |
| Heterogeneity | 0 | 0 | 0 | 39% |
| χ^2^ | 1.64 | | 0.05 | |
| *P*-value | 0.20 | | 0.82 | |
| excellent outcomes (mRS score:0-1) | | | | |
| No of studies | 8 | 8 | 4 | 4 |
| OR and 95% CI | 1.12 (0.87-1.45) | 1.46 (1.14-1.85) | 1.78 (1.28-2.47) | 1.39 (1.11-1.74) |
| Heterogeneity | 50% | 69% | 0 | 29% |
| χ^2^ | 2.14 | | 1.48 | |
| *P*-value | 0.14 | | 0.22 | |
| SR | | | | |
| No of studies | 27 | 11 | 5 | 4 |
| OR and 95% CI | 1.17 (0.94-1.46) | 1.35 (1.10-1.64) | 1.07 (0.51-2.23) | 1.33 (1.17-1.51) |
| Heterogeneity | 62% | 27% | 86% | 0 |
| χ^2^ | 0.80 | | 0.33 | |
| *P*-value | 0.37 | | 0.57 | |
| aICH | | | | |
| No of studies | 15 | 8 | 4 | 2 |
| OR and 95% CI | 1.21 (0.95-1.55) | 1.30 (0.98-1.72) | 1.03 (0.53-1.98) | 1.05 (0.83-1.35) |
| Heterogeneity | 51% | 325 | 47% | 29% |
| χ^2^ | 0.15 | | 1.48 | |
| *P*-value | 0.70 | | 0.22 | |
| Mortality | | | | |
| No of studies | 21 | 13 | 8 | 6 |
| OR and 95% CI | 0.66 (0.56-0.82) | 0.67 (0.53-0.83) | 0.95 (0.68-1.34) | 0.58 (0.43-0.78) |
| Heterogeneity | 40% | 51% | 41% | 65% |
| χ^2^ | 0.01 | | 4.65 | |
| *P*-value | 0.92 | | 0.03 | |

**Supplemental Table 4** Assessment of quality using the Newcastle-Ottawa quality assessment scale (observational study)

| Studies | Selection | | | | Comparability | Outcome | | | Score |
| --- | --- | --- | --- | --- | --- | --- | --- | --- | --- |
|  | 1 | 2 | 3 | 4 | 1 | 1 | 2 | 3 |  |
| Dávalos, et al (2012) | * | * | * |  | * | * | * | * | 7 |
| Pfefferkorn, et al (2012) | * | * | * |  | ** | * | * | * | 8 |
| Kass-Hout, et al (2014) | * | * | * |  | ** | * | * | * | 8 |
| Guedin, et al (2015) | * | * | * | * | ** | * | * |  | 8 |
| Leker, et al (2015) | * | * | * | * | ** | * | * |  | 8 |
| Broeg-Morvay, et al (2016) | * | * | * | * | ** | * | * |  | 8 |
| Behme, et al (2016) | * | * | * | * | ** | * | * |  | 8 |
| Minnerup, et al (2016) | * | * | * | * | ** | * | * |  | 8 |
| Froehler, et al (2017) | * | * | * | * | ** | * | * |  | 8 |
| Alonso de Leciñana, et al (2017) | * | * | * | * | ** | * | * | * | 9 |
| Weber, et al (2017) | * | * | * |  | ** | * | * |  | 7 |
| Merlino, et al (2017) | * | * | * | * | ** | * | * |  | 8 |
| Bellwald, et al (2017) | * | * | * |  | ** | * | * | * | 8 |
| Abilleira, et al (2017) | * | * | * | * | * | * | * | * | 8 |
| Bourcier, et al (2017) | * | * | * |  | ** | * | * | * | 8 |
| Maier, et al (2017) | * | * | * | * |  | * | * | * | 7 |
| Wang, et al (2017) | * | * | * |  | ** | * | * | * | 8 |
| Wee, et al (2017) | * | * | * |  | ** | * | * | * | 8 |
| Park, et al (2017) | * | * | * | * | ** | * | * | * | 9 |
| Ferrigno, et al (2018) | * | * | * | * | ** | * | * |  | 8 |
| Choi, et al (2018) | * | * | * | * | ** | * | * |  | 8 |
| Al-Khaled, et al (2018) | * | * | * |  | * | * | * | * | 8 |
| Gong, et al (2018) | * | * | * |  | ** | * | * | * | 8 |
| Imbarrato, et al (2018) | * | * | * |  |  | * | * | * | 6 |
| Leker, et al (2018) | * | * | * | * | ** | * | * |  | 8 |
| Rai, et al (2018) | * | * | * |  | * | * | * | * | 7 |
| Sallustio, et al (2018) | * | * | * |  | ** | * | * | * | 8 |
| Heinrichs, et al (2018) | * | * | * | * | ** | * | * |  | 8 |
| Di Maria, et al (2018) | * | * | * |  | ** | * | * | * | 8 |
| Goyal, et al (2019) | * | * | * |  | ** | * | * | * | 8 |
| Guimarães Rocha, et al (2019) | * | * | * | * | ** | * | * | * | 9 |
| Balodis, et al (2019) | * | * | * | * | * | * | * | * | 8 |
| Maingard, et al (2019) | * | * | * |  | ** | * | * | * | 8 |
| Casetta, et al (2019 | * | * | * |  | ** | * | * | * | 8 |
| Hassan, et al (2019) |  |  |  |  |  |  |  |  |  |
| Reiff, et al (2020) | * | * | * |  | * | * | * | * | 7 |
| Yi, et al (2020) | * | * | * | * | ** | * | * |  | 8 |
| Hinsenveld, et al (2020) | * | * | * | * | ** | * | * |  | 8 |
| Jian, et al (2021) | * | * | * | * | ** | * | * | * | 9 |
| Kandregula, et al (2021) | * | * | * |  | * | * | * | * | 8 |
| Tong, et al (2021) | * | * | * | * | ** | * | * | * | 9 |
| Zha, et al (2021) | * | * | * | * | ** | * | * |  | 8 |
| Machado, et al (2021) | * | * | * |  | * | * | * | * | 7 |
| Platko, et al (2022) | * | * | * |  | * | * | * | * | 7 |
| Dicpinigaitis, et al (2022) | * | * | * |  | ** | * | * |  | 7 |

**Supplemental Table 5** The comparison with prior meta-analysis

| Study  (Author, year) | populations | NO. of studies | Studies design | NO. of Outcomes | Adjusted data |
| --- | --- | --- | --- | --- | --- |
| Katsanos et.al 2019 | 11,798 | 38 | OS | (1) mRS score: 0-2  (2) successful recanalization  (3) mortality at 3 months or discharge  (4) sICH and aICH  (5) onset to groin puncture time  (6) groin puncture to reperfusion time | Yes |
| Vidale et.al 2020 | 9,117 | 35 | RCTs and OS | (1) mRS score:＜3  (2) successful recanalization  (3) mortality at 90 days  (4) sICH | NO |
| Vidale et.al 2021 | 1,657 | 5 | RCTs | (1) mRS score:＜3  (2) successful recanalization  (3) mortality at 90 days  (4) sICH | NO |
